# Mis-localization of endogenous TDP-43 leads to ALS-like early-stage metabolic dysfunction and progressive motor deficits

**DOI:** 10.1186/s13024-024-00735-7

**Published:** 2024-06-20

**Authors:** Yiying Hu, Alexander Hruscha, Chenchen Pan, Martina Schifferer, Michael K. Schmidt, Brigitte Nuscher, Martin Giera, Sarantos Kostidis, Özge Burhan, Frauke van Bebber, Dieter Edbauer, Thomas Arzberger, Christian Haass, Bettina Schmid

**Affiliations:** 1 German Center for Neurodegenerative Diseases (DZNE) Munich, Munich, Germany; 2 Metabolic Biochemistry, Biomedical Centre (BMC), Faculty of Medicine, Ludwig-Maximilian University, Munich, Germany; 3 Munich Medical Research School (MMRS), Munich, Germany; 4 Neurology Clinic and National Center for Tumor Diseases, Heidelberg University Hospital, Heidelberg, Germany; 5 Clinical Cooperation Unit Neurooncology, German Cancer Consortium (DKTK), German Cancer Research Center (DKFZ), Heidelberg, Germany; 6 Munich Cluster for Systems Neurology (SyNergy), Munich, Germany; 7 Zentrum Für Neuropathologie, Ludwig-Maximilians University, Munich, Germany; 8 Leiden University Medical Center, Leiden, Netherlands; 9 Department of Psychiatry and Psychotherapy, University Hospital, Ludwig-Maximilians University, Munich, Germany

**Keywords:** ALS, TDP-43, Animal model, Neurodegeneration, Metabolic dysfunction, Hypothalamus, Zebrafish

## Abstract

**Background:**

The key pathological signature of ALS/ FTLD is the mis-localization of endogenous TDP-43 from the nucleus to the cytoplasm. However, TDP-43 gain of function in the cytoplasm is still poorly understood since TDP-43 animal models recapitulating mis-localization of endogenous TDP-43 from the nucleus to the cytoplasm are missing.

**Methods:**

CRISPR/Cas9 technology was used to generate a zebrafish line (called CytoTDP), that mis-locates endogenous TDP-43 from the nucleus to the cytoplasm. Phenotypic characterization of motor neurons and the neuromuscular junction was performed by immunostaining, microglia were immunohistochemically localized by whole-mount tissue clearing and muscle ultrastructure was analyzed by scanning electron microscopy. Behavior was investigated by video tracking and quantitative analysis of swimming parameters. RNA sequencing was used to identify mis-regulated pathways with validation by molecular analysis.

**Results:**

CytoTDP fish have early larval phenotypes resembling clinical features of ALS such as progressive motor defects, neurodegeneration and muscle atrophy. Taking advantage of zebrafish’s embryonic development that solely relys on yolk usage until 5 days post fertilization, we demonstrated that microglia proliferation and activation in the hypothalamus is independent from food intake. By comparing CytoTDP to a previously generated TDP-43 knockout line, transcriptomic analyses revealed that mis-localization of endogenous TDP-43, rather than TDP-43 nuclear loss of function, leads to early onset metabolic dysfunction.

**Conclusions:**

The new TDP-43 model mimics the ALS/FTLD hallmark of progressive motor dysfunction. Our results suggest that functional deficits of the hypothalamus, the metabolic regulatory center, might be the primary cause of weight loss in ALS patients. Cytoplasmic gain of function of endogenous TDP-43 leads to metabolic dysfunction in vivo that are reminiscent of early ALS clinical non-motor metabolic alterations. Thus, the CytoTDP zebrafish model offers a unique opportunity to identify mis-regulated targets for therapeutic intervention early in disease progression.

**Supplementary Information:**

The online version contains supplementary material available at 10.1186/s13024-024-00735-7.

## Background

Amyotrophic lateral sclerosis (ALS) and frontotemporal lobar degeneration (FTLD) can share common clinical features, including motor deficits, dementia, weight loss and metabolic dysfunction [1, 2]. In addition, ALS and FTLD are characterized by the abnormal aggregation of the TAR DNA-binding protein of 43 kDa (TDP-43, TARDBP). Inclusions of TDP-43 are found in ~ 97% ALS and ~ 45% FTLD patients [3]. A striking feature of these aggregates is that TDP-43 is abnormally phosphorylated, proteolytically processed, ubiquitinated and predominantly localized to the cytoplasm [4]. TDP-43 is a DNA and RNA binding protein that is highly conserved, widely expressed and mainly localized in the nucleus [5–7]. TDP-43 plays important roles in splicing regulation, transcription, RNA transport, RNA stability and microRNA processing. However, its physiological role in ALS/FTLD pathophysiology is only poorly characterized [8].

Researchers have developed numerous animal models for ALS to gain insights into the molecular mechanisms of disease [9]. Most of these animal models are based on introducing genetic mutations found in familial ALS or overexpression of mutant TDP-43 [9, 10]. However, familial ALS accounts for only ~ 10% of all ALS cases and might not fully recapitulate the etiology of sporadic ALS cases [2]. Moreover, TDP-43 function is very dose sensitive, accordingly endogenous TDP-43 levels are tightly regulated by an autoregulatory negative feedback loop. High amounts of TDP-43 protein reduce TDP-43 RNA levels by binding to its own 3' UTR and destabilizing the mRNA [11–13]. It has also been shown that overexpression of TDP-43 is toxic to neurons [13]. Both approaches therefore only poorly recapitulate disease conditions in animal models.

The hallmark features of TDP-43 proteinopathies are the clearance of endogenous TDP-43 from the nucleus with aggregation in the cytoplasm [3]. Currently no vertebrate animal model recapitulates the shift of endogenous TDP-43 to the cytoplasm. Utilizing CRISPR/Cas9 technology, we now modified the nuclear localization sequence 1 (NLS1) of endogenous Tardbp in zebrafish, leading to its mis-localization from the nucleus to the cytoplasm. We and others have previously shown that in the absence of Tardbp function the second TDP-43 orthologue in zebrafish, the Tar DNA binding protein like (Tardbpl), can fully compensate Tardbp function through altering its splicing in favor of a full length Tarbpbl_tv1 transcript variant [14, 15]. Therefore, analysis of altered Tardbp function can only be revealed in a Tardbpl KO background in zebrafish. In the absence of Tardbpl function, cytoplasmic mis-localization of endogenous Tardbp resulted in progressive motor deficits, disruption of the neuromuscular junction, neurodegeneration, muscle atrophy, microglial activation in the hypothalamus and metabolic dysfunction. Comparing these findings with TDP-43 double knock-out (TDP DKO; *tardbp − / − ; tardbpl − / −)* phenotypes, we uncovered that mis-localization of Tardbp, rather than loss of Tardbp nuclear function, leads to metabolic dysfunction. The early onset of phenotypes in our novel TDP-43 animal model (called CytoTDP) will allow the study of early metabolic alterations in ALS and serve as a valuable tool for preclinical investigations of metabolic dysregulation associated with ALS.

## Methods

### Fish maintenance

All fish were housed at the fish facility of the German Center for Neurodegenerative Diseases (DZNE) in Munich according to local animal welfare regulations. All procedures were carried out with approval and according to the regulations of the District Government of Upper Bavaria, Germany. Larvae were obtained by natural spawning and raised at 28.5°C in incubators in E3 medium until 5 days post fertilization (dpf). Larvae older than 5 dpf were kept in the cocultures with rotifers until 16 dpf [16–18]. Larvae were staged according to Kimmel et al. [19].

### Generation of genome edited fish

Generation and breeding of edited fish lines was approved by the government of upper Bavaria. ΔNLS-Tardbp line was generated by injection of a donor construct (ssDNA: tgtgtatatatagcaattgagtttttcttttctagAAACTGTTCTGCCAGATAATgcagcagcaATGGATGAGATCGATGCTTCATCTGCGACCAAGATCAAGAGAGGAGATCAGAAGAC), Cas9 protein (IDT Alt-R® S.p. Cas9 Nuclease V3) and a crRNA:tracrRNA duplex (IDT, crRNA sequence: CTGTTCTGCCAGATAATAAG, targeting the NLS of the *tardbp* gene (Cas9 1.5 µg/µl, 1.5 µM gRNA and 100 mM donor DNA). Injected fish were raised to adulthood and finclips were analyzed by PCR and restriction length polymorphism (RFLP) analysis for successful DNA integration (forward primer T30: ccttctgaattcttttagctgtcca; reverse primer T31: GCACCATGATGACTTCCCCA; restriction enzyme used for RFLP: ApeKI, NEB) as described [20]. In brief, we injected Cas9 protein, guide RNAs targeting the NLS1 and a single stranded DNA donor template containing the NLS1 mutation into fertilized one-cell stage embryos and raised the injected fish to adulthood. Potential successful founder fish were fin clipped and prescreened for integration of the donor template by PCR. Only P0 positive fin clip founders were selected to screen for germline transmission. Positive founder fish were outcrossed and the offspring was used to screen for germ line transmission followed by sequencing of the targeted region over the junctions of the donor construct. We obtained 4 positive F1 fish from 60 prescreened founder fish corresponding to a germline transmission rate of 6,6%. Successful mutation of NLS1 was confirmed by Sanger sequencing (SFigure 1).

### Immunohistochemistry and immunofluorescence

Zebrafish embryos were fixed using freshly prepared 4% paraformaldehyde (PFA) in phosphate-buffered saline (PBS) for 36 h at 4 °C. After fixation, the embryos were thoroughly washed with PBS and embedded in paraffin. Embryo paraffin blocks were cut into 2 μm thick sections. Immunhistochemistry for TDP-43 (Tardbp 30G5-1–1, epitope: MDSKSSGWGM, mouse IgG3, diluted 1:50 in Roche diluens) and phosphorylated TDP-43 (Ser409/410, proteintech, 80007–1-RR, diluted 1:500) were performed without hematoxylin counterstaining in a Ventana BenchMark ULTRA (Roche) using the ultraView diaminobenzidine (DAB) detection kit according to the instructions of the manufacturer. Incubation time and temperature of the primary antibody was 32 min at 37 °C. Immunofluorescence for TDP-43 (Tardbp 4A12-11111, epitope: TSTSGTSSSRDQAQTY, rat IgG2a, diluted 1:1) required 30 min boiling in Tris–EDTA (pH 9) for antigen retrieval and was performed with DAPI (Sigma-Aldrich, D8417, 1:1000) for nuclei staining in blocking solution (5% goat serum, 5% BSA and 0.1% Triton-X). Goat anti-rat Alexa Fluor antibody (1:400) was used as secondary antibody.

### Western blotting

Embryos or larvae were frozen in liquid nitrogen and 20–40 μl of 4 × Lämmli buffer were added per fish. Fish were lysed by sonication. Lysates were boiled for 5 min at 95 °C while shaking at 800 rpm. Supernatant was loaded after a 5 min spin at 13,000 rpm at room temperature. A total of 10–20 μl of supernatant per lane were loaded on 12% (wt/vol) Tris glycine gels. After electrophoresis, proteins were transferred to PVDF membranes (Millipore). Membranes were blocked for 1 h in PBS with 0.1% Tween 20 (PBST) with 0.2% I-Block (Invitrogen™, T2015). The primary antibody was incubated in blocking solution overnight at 4 °C. We performed Western blotting using the following primary antibodies: anti-ubiquitin antibody (#04–263 clone FK2, Merck), dilution 1:40,000; α-tubulin (Sigma T6199), dilution 1:10,000. The following antibodies were generated by the Institute of Molecular Immunology, Helmholtz Center Munich by standard procedures: Tardbpl_tv1 16C8-11 (Tardbpl_tv1 epitope: SRQMMDRG- RFGGYG), dilution 2:5, rat IgG2a; Tardbp 4A12-111 (Tardbp epitope: TSTSGTSSSRDQAQTY), dilution 1:1, rat IgG2a; Tardbp 30G5-1–1 (Tardbp epitope: MDSKSSGWGM), dilution 1:20, mouse IgG3, phosphoTDP-43 Ser 409/410 (1D3), dilution 1:10. After washing 4 × 15 min with PBST with 0.2% I-Block (Invitrogen™, T2015), the secondary antibody was incubated for 1 h in PBST with blocking solution. We performed Western blotting using the following secondary antibodies: Anti-rabbit IgG, HRP conj. (Promega, W4011), dilution 1:10,000; Anti-mouse IgG, HRP conj. (Promega, W4021), dilution 1:5,000. The following antibodies were generated by the Institute of Molecular Immunology, Helmholtz Center Munich by standard procedures: Anti-rat IgG2a, HRP conj., dilution 1:1,000 Anti-mouse IgG3, HRP conj., WB 1:1,000. Development of the membrane after 6 × 15 min PBST washes was performed with ECLplus (Amersham).

### Tissue clearing and whole-mount immunofluorescence

Zebrafish larvae from the Tg(mpeg1.1:EGFP-CAAX) transgenic line were collected at 5 dpf and 8 dpf respectively and fixed with 4% PFA at 4 °C for 24 h. After a few short washes with PBS, a modified protocol derived from DEEP-Clear [21] was performed as follows. In brief, specimens were treated with prechilled acetone at -20 °C overnight and washed three times with PBS at room temperature (RT) with gentle shaking. Then, depigmentation was conducted with 3% H_2_O_2_ (neoLab, LC-4458.2) prepared in 0.8% KOH (Sigma-Aldrich, 221473) for 5–10 min, depending on the sample size. The depigmentation was carefully monitored under a dissection microscope (Leica S4E, KL200 LCD), and the tubes containing the specimens were manually rotated to avoid accumulation of bubbles. After five short PBS washes, the specimens were treated with Solution-1.1 (10% (v/v) N,N,N′,N′-Tetrakis-(2-hydroxyethyl)-ethylendiamin (Sigma-Aldrich, 87600-100ML), 5% (w/v) urea (Carl Roth, 3941.1) and 5% (v/v) Triton X-100 (Sigma-Aldrich, X100-1L) in dH_2_O) for 5–10 min at RT for further depigmentation and permeabilization. After five short PBS washes, the blocking solution containing 10% goat serum (Gibco, 16210072) in PBS was applied at RT with gentle shaking for 3 h and the specimens were then incubated in primary antibody solution with 5% goat serum in PBS for 5 days at 4 °C with gentle shaking. Specifically, rabbit anti-GFP antibody (ThermoFisher, A-11122, 1:200 dilution), rat anti-5-HT antibody (Sigma-Aldrich, MAB352, 1:200 dilution), mouse monoclonal antibody ZNP-1 (DSHB, 1:100 dilution) were used in this study. Following multiple PBS washes at RT, the secondary antibodies were incubated with goat anti-rabbit IgG Alexa 488 (ThermoFisher, A-11034, 1:200 dilution), goat anti-rat IgG Alexa 546 (ThermoFisher, A-11081, 1:200 dilution), goat anti-rat IgG Alexa 633 (ThermoFisher, A-21094, 1:200 dilution), donkey anti-mouse IgG Alexa 647 (ThermoFisher, A-31571, 1:200 dilution), together with propidium iodide (Sigma-Aldrich, P4864, 1:100) or DAPI (Sigma-Aldrich, D8417, 1:100) for nuclei staining in 5% goat serum in PBS for 5 days at 4 °C. For samples further labeled with TUNEL, In Situ Cell Death Detection Kit TMR red (Roche, 12156792910) was applied after the secondary antibodies incubation, and the staining solution was prepared by adding 5 µl enzyme solution and 45 µl labeling solution into 1 ml PBST. Following 3 short washes in PBS, samples were incubated in TUNEL staining solution at 37 °C for 2.5–3 h with 200 rpm horizontal shaking. After two short washes in PBST at RT, the samples were further processed.

After immunofluorescence or TUNEL staining and five short PBS washes, the specimens were incubated in 1% agarose gel and carefully aligned ventral side up before solidification. Agarose gel blocks containing individual specimens were then cut and collected in 2 ml Eppendorf tubes. Dehydration was conducted by gradient incubation of 20%, 40%, 60%, 80% of methanol (Carl Roth, 4627.1) solutions in dH_2_O (1 h for each step) and overnight incubation of 100% methanol at 4 °C with gentle shaking. The refractive index matching was performed in BABB, a 1:2 mixture of benzyl alcohol (Sigma, 24122) and benzyl benzoate (Sigma, W213802). The specimens were covered with aluminum foil to prevent potential fluorescent bleaching from the secondary antibody incubation. Once optical transparency was achieved, the specimens were processed with further microscopic assessment.

### Microglia and TUNEL quantification by confocal microscopy

A glass bottom petri dish (ibidi, 81156) containing cleared agarose gel blocks with specimens were mounted on an inverted confocal microscope (Leica TCS SP5 II or Zeiss LSM 710 ConfoCor 3). The entire heads of the zebrafish samples were scanned using a 10 × air objective with a step size of 5 μm, and an additional 4 × zoom was applied to image the hypothalamic region with a step size of 2 μm. The scan files were then loaded in FIJI with ‘Split channels’ option, and scans of each channel were saved as separate tiff files. To compare and quantify the microglia cell number in whole-brain and in hypothalamic region, the ‘Synchronize Windows’ function was applied with the GFP channel and the nuclei staining channel files. The border of the brain tissue was carefully characterized by the nuclei staining channel. Only the GFP positive cells in brain tissue were labeled manually with ‘Multi-point’ function and added to the ‘ROI Manager’. After labeling all the brain specific GFP positive cells, ‘Measure’ was applied to calculate the number of microglia. The same strategy was applied for TUNEL quantification. To analyze the morphology of microglia, high resolution confocal images of the hypothalamus were loaded in FIJI. Parameters (microglial somata area, number of main processes and average process length) were measured and quantified. Overall, 50 cells from 10 zebrafish were analyzed in each group.

### Whole-mount neuromuscular junctions staining

Larvae were fixed in 4% (wt/vol) PFA overnight at 4℃. Larvae were washed three times 10 min in PBS and permeabilized by incubating in acetone overnight at -20℃, followed by two 5 min PBS washes. Larvae at 8 and 16 dpf were incubated for 40 min in 2 mg/mL collagenase, then were washed twice for 10 min with PBS. Larvae were blocked for 40 min in blocking solution (10% newborn calf serum, 1% DMSO, 0.8% Triton X-100, and PBST, pH 7.4) and then incubated overnight in the mouse monoclonal antibody ZNP-1 (DSHB, 1:100 dilution) and Alexa Fluor 488-α-bungarotoxin (Molecular Probes B-13422, 1:100 dilution) in blocking solution for 2 nights at 4℃. Larvae were then washed three times 10 min in blocking solution and incubated in goat anti-mouse Alexa Fluor antibody (1:200) in blocking solution for 2 nights at 4 °C, then washed for three times 10 min with PBS. ZNP-1 antibody detecting synaptotagmin 2 (Developmental Studies Hybridoma Bank—DSHB) was used as a presynaptic marker and α-bungarotoxin was used as a post-synaptic nicotinic acetylcholine receptor (nAChR) marker for the characterization of the neuromuscular junction (NMJ).

### Confocal microscopy and NMJ analysis

The larvae (8 dpf and 16 dpf) were mounted on a glass bottom petri dish (ibidi, 81156) in 1.5% (w/v) low melting point agarose (MetaPhor™ Agarose, 50180). Larvae were gently moved to align the sagittal plane parallel to the glass slide during mounting. Larvae were imaged using an inverted Zeiss LSM800 confocal laser scanning microscope (Carl Zeiss) with a 10 × objective. NMJ quantifications and co-localization analysis were measured using ImageJ 2.3.0. The ‘colour threshold’ and ‘measure’ function was applied for analysis.

### Motor neuron staining and quantification

Larvae were fixed in 4% (wt/vol) PFA overnight at 4 °C. Larvae were washed three times 10 min in PBST and transferred to a 25% sucrose in PBS solution until they sink to the bottom. The larvae were then transferred to a 30% sucrose in PBS solution until they sink to the bottom. They were then mounted into molds in Tissue-Tek O.C.T compound (Sakuraus, 4583) and frozen on dry ice. 20μm sections were cut with a CryoStar NX70 (Epredia) and slices were collected on Superfrost Plus slides (Epredia, J1800AMNZ).

For the ChAT antibody staining of the cryosections an established protocol [22] was slightly modified. Cryosections were stored at -20 °C and air-dried for 3 h before staining. After two PBST washes, cryosections were incubated for 20 min in 2% H_2_O_2_/PBS and subsequently rinsed twice in PBDT (1 × PBS containing 0.2% Tween-20 & 1% DMSO) before 3 × 5 min PBDT washes. Cryosections were blocked in 10% NDS (normal donkey serum; Santa Cruz Biotechnology, Santa Cruz, CA, USA) in PBDT at RT for at least 1 h before incubation with the primary antibody (ChAT antibody; Chemicon AB144P, Merck, Darmstadt, Germany; 1:100 in 10% NCS in PBDT) for at least 18 h at 4 °C followed by 1 h at RT. After removal of the primary antibody, cryosections were rinsed and then washed 6 × 5 min with PBDT before incubation with the secondary antibody (Alexa488-conjugated donkey anti-goat A-11055; Thermo Fisher Scientific; 1:250 in 10% NCS in PBDT) at RT for at least 1.5 h. After removal of the secondary antibody, cryosections were rinsed and then washed 6 × 5 min with PBDT before mounting with 2 drops of VECTASHIELD mounting medium containing DAPI (Vector Laboratories, Vec-H-1500). Pictures were taken using the Axio Scope.A1 (Carl Zeiss) for manual quantification of motor neurons and the confocal microscope LSM800 for the generation of representative images in Fig. 5A (Carl Zeiss). Sections from 6 animals per group were used for quantification.

### Scanning electron microscopy

Fish larvae were anesthetized and fixed (4% PFA and 2.5% glutaraldehyde in 0.1 M sodium cacodylate buffer, pH 7.4; Science Services) in a laboratory microwave (PELCO BioWave). The tail and head were dissected, and the remaining larvae immersion fixed for 5 days. We applied a standard rOTO microwave staining protocol [23] including postfixation in 2% osmium tetroxide (EMS), 1.5% potassium ferricyanide (Sigma) in 0.1 M sodium cacodylate (Science Services) buffer (pH 7.4). Staining was enhanced by reaction with 1% thiocarbohydrazide (Sigma) for 45 min at 40 °C. The tissue was washed in water and incubated in 2% aqueous osmium tetroxide, washed and further contrasted by overnight incubation in 1% aqueous uranyl acetate at 4 °C and 2 h at 50 °C. Samples were dehydrated in an ascending ethanol series and infiltrated with LX112 (LADD). Blocks were cured for 48 h, trimmed (TRIM2, Leica) and sectioned at 100 nm thickness using a 35° ultra-diamond knife (Diatome) on an ultramicrotome (UC7, Leica). Sections were collected onto 1 × 0.5 cm carbon nanotube tape strips (Science Services) for scanning EM (SEM) acquisition. The samples on tape were attached to adhesive carbon tape (Science Services) on 4-inch silicon wafers (Siegert Wafer) and grounded by adhesive carbon tape strips (Science Services). EM micrographs were acquired on a Crossbeam Gemini 340 SEM (Zeiss) with a four-quadrant backscatter detector at 8 kV using ATLAS5 Array Tomography (Fibics). Medium lateral resolution images (100 nm) allowed the identification of regions of interest that were in turn imaged at 4–10 nm lateral resolution. Image analysis was performed in Fiji [24].

### Behavioral assays

For the touch-evoked escape response assay, a total of 30 larvae (1.5 dpf) were dechorionated manually 1 h prior analysis. To assess the escape response, fish tails were gently touched at least 3 times with the tip of a fine needle. To quantify percentage of normal response, an escape response where the fish did not move a distance equivalent to at least one time its body length was considered reduced. Percentage of normal response in CytoTDP and their siblings was quantified.

For the locomotion assay, a total of 94 larvae (each embryo individually placed in each well) had their activity automatically tracked using the Zebrabox tracking device at 5 dpf and 8 dpf (Viewpoint, ZebraLabv3). Recordings were made directly on the 24-well plates. Distances and time moving were recorded for 60 min. A transparent background mode with a detection threshold of 18 was set. Behavioral endpoints measured were swimming distance and movement time.

### RNA isolation and library preparation

1.5 dpf larvae (*n* = 3 per sample per group) were frozen in liquid nitrogen and then were homogenized in QIAzol using homogenizer (KONTES®). Total RNA was isolated using miRNeasy Micro Kit (QIAGEN) following the manufacturer’s instructions. RNA quantity and quality were controlled on Agilent 2100 BioAnalyzer. Only RNAs with a RIN value = 10 were used for totalRNA sequencing (Ribo-depleted). The libraries were prepared using the TruSeq stranded total RNA Sample Preparation kit (Illumina), following the kit's instructions. After a final QC, the libraries were pooled and sequenced in a paired-end mode (2 × 150 bases) in the Novaseq6000 sequencer (Illumina) with a depth of ≥ 120 million reads per sample. Raw fastq files were checked using FastQC 1.9. The data was mapped to the Zebrafish genome and gene annotations were obtained from Ensembl (GRCz11). 5 dpf larvae (*n* = 10 per sample) were frozen in liquid nitrogen and total RNA was extracted using the miRNeasy Micro Kit (QIAGEN) following the manufacturer’s instructions. RNA quality was tested on a Agilent 2100 BioAnalyzer. Only RNAs with a RIN value ≥ 9.9 were used for mRNA sequencing. Library preparation was performed using a strand-specific mRNA library (BGI Gemonics). The sequencing was performed in a paired-end mode (2 × 150 bases) in DNBSEQ G400 with a depth of ≥ 60 million reads per sample. Raw fastq files were checked using SOAPnuke 1.5.6. The data was mapped to the zebrafish genome (GRCz11).

### Data analysis

DESeq2 pipeline in R environment was used for differential expression analysis based on raw counts [25]. The cutoff for the differentially regulated genes was based on adjusted p value equivalent to the 5% false discovery rate (padj ≤ 0.05), according to the DESeq2 pipeline. Gene ontology analysis was done using the clusterProfiler package in R, only groups with more than four significant genes were analyzed. Heatmap was done using the pheatmap package in R. Hubgene analysis was done by Cytoscape plugin cytoHubba [26].

### Glycogen quantification

5 dpf larvae were frozen in liquid nitrogen (*n* = 5 per sample per group) and were homogenized rapidly in chilled water using a homogenizer (KONTES®). Glycogen was quantified with the Glycogen Assay Kit II (colorimetric) (Abcam, ab169558) according to the manufacturer’s instructions.

### Statistics

Statistical analyses were performed using GraphPad Prism 9.3.0. Mantel-Cox test was used for survival analysis. Data distributions were checked for normality by the Shapiro–Wilk test. When variables were normally distributed, we used two-tailed T tests to compare two groups, and ANOVAs to compare more than two groups. When variable is not normally distributed, we used Mann–Whitney tests to compare two groups, and Kruskal–Wallis tests to compare more than two groups.

## Results

### Generation of the ΔNLS-Tardbp mutation in zebrafish

**Fig. 1:**
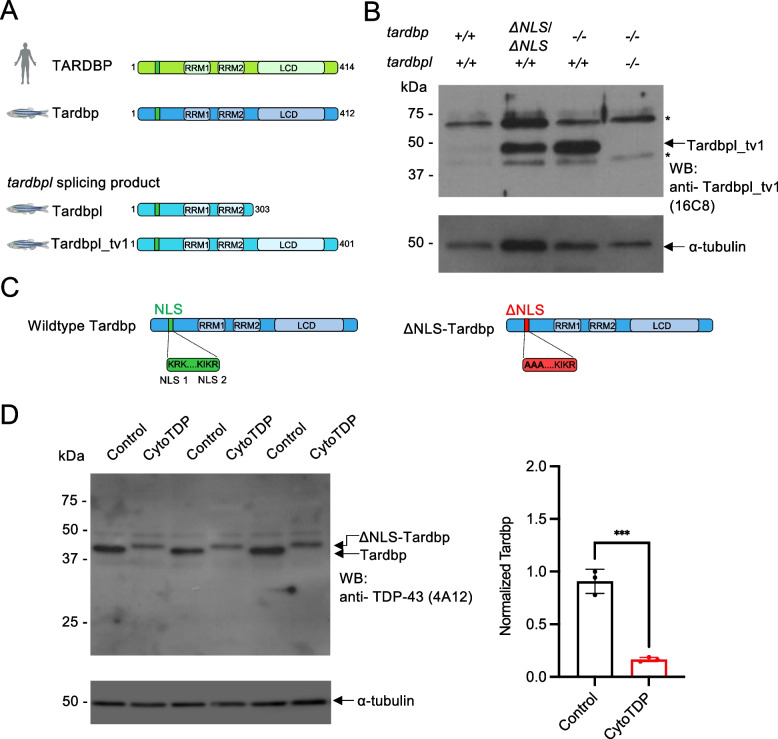
Biochemical characterization of ΔNLS-Tardbp fish. **A** Schematic drawing shows splicing products from zebrafish 2 orthologs of human *TARDBP*, *tardbp* and *tardbpl*. Zebrafish Tardbp has 2 RNA recognition motifs (RRM1 and RRM2) and a low-complexity domain (LCD). Tardbpl and Tardbpl_tv1 are splicing products from the *tardbpl* gene. While zebrafish Tardbpl does not have a low-complexity domain, zebrafish Tardbpl_tv1 has 2 RNA recognition motifs (RRM1 and RRM2) and a low-complexity domain. **B** Western blot analysis with the Tardbpl_tv1 specific antibody 16C8 detects Tardbpl_tv1 levels to be upregulated in *tardbp ΔNLS/ΔNLS* and *tardbp -/-* embryos compared to wildtype embryos. TDP DKO larvae in lane 4 serve as a control for the specificity of the Tardbpl_tv1 antibody. Asterics mark unspecific bands. α-tubulin serves as a loading control. **C** Schematic drawing highlights bipartite nuclear localization sequence (NLS) in zebrafish Tardbp. The ΔNLS-Tardbp mutation was generated in NLS1 by mutating the amino acids KRK to AAA. **D** Western blot analysis of 3 biological replicates with the Tardbp antibody 4A12 detects reduced Tardbp levels and a band shift to a higher molecular weight in CytoTDP embryos compared to Control (*tardbp* + / + ; *tardbpl* -/-) embryos. Semi-quantitative analysis of the Western blots revealed that the Tardbp levels normalized to α-tubulin in 5 dpf CytoTDP embryos (*n* = 3) are only 18% compared to Control (*n* = 3) (***p = 0.0004, Unpaired T test)

Zebrafish has 2 orthologs of human *TARDBP*, referred to as *tardbp* and *tardbpl*. The *tardbp* gene encodes for Tardbp (Tar DNA binding protein of 43 kDa, 412-aa), which is highly homologous to human TDP-43. In contrast, the *tardbpl* gene is spliced to Tardbpl (Tar DNA binding protein of 43 kDa-like, 303-aa) that lacks the low-complexity domain, as well as to Tardbpl_tv1 (*tardbpl* transcript variant 1, 401-aa), which contains a low-complexity domain (Fig. 1A) [14, 15]. In wildtype fish, only very low amounts of Tardbpl_tv1 level are expressed (Fig. 1B). However, we and others have previously shown that in Tardbp KO zebrafish, *tardbpl* splicing is changed in favor of Tardbpl_tv1 to compensate loss of Tardbp function (Fig. 1B) [14, 15]. Both parts of the bipartite nuclear localization sequence (NLS) in TDP-43 are required for nuclear localization (Fig. 1C, SFigure 1). Thus, mutations in either NLS motif lead to TDP-43 accumulation in the cytoplasm [27]. To achieve endogenous Tardbp cytoplasmic translocation, we generated a ΔNLS-Tardbp knockin line by mutating the zebrafish Tardbp NLS 1 amino acids KRK to AAA (Fig. 1C, SFigure 1) by homology repair mediated CRISPR/Cas9 genome editing (SFigure 1).

Since complete loss of Tardbp function has no morphological phenotype due to functional compensation through Tardbpl_tv1 [14, 15], we hypothesized that Tardbpl_tv1 can also compensate a potential homozygous ΔNLS-Tardbp phenotype. Thus, we generated two zebrafish lines: 1. *tardbp ΔNLS/ΔNLS; tardbpl* + */* + . and 2. *tardbp ΔNLS/ΔNLS; tardbpl -/-*. We first analyzed *tardbp ΔNLS/ΔNLS*; *tardbpl* + */* + fish and found that they looked phenotypically like wildtype and are viable (SFigure 2A,B). We next tested the hypothesis if Tardbpl_tv1 is upregulated in *ΔNLS/ΔNLS*; *tardbpl* + */* + fish by using a specific antibody against Tardbpl_tv1 for Western blot analysis. Compared to wildtype fish, the Tardbpl_tv1 level were increased in homozygous ΔNLS-Tardbp and also upregulated in Tardbp knockout zebrafish as previously shown, suggesting that *tardbpl* rescues a ΔNLS-Tardbp phenotype by shifting splicing to the functionally equivalent Tardbpl_tv1 variant (Fig. 1B). Thus, we next analyzed the *tardbp ΔNLS/ΔNLS* mutation in a *tardbpl -/-* background. For convenience and brevity, we called the *tardbp ΔNLS/ΔNLS; tardbpl -/-* larvae CytoTDP and the *tardbp* + */* + *; tardbpl -/-* larvae Control. Using a Tardbp specific antibody, we discovered that in CytoTDP fish ΔNLS-Tardbp shifted from about 43 kDa to a higher molecular weight, while the overall protein level decreased by 82% (Fig. 1D). We used 2 different antibodies (SFigure 3A) on Control and CytoTDP larvae from 2 to 5 dpf to validate our results and to confirm the specificity of the Tardbp band in CytoTDP fish (SFigure 3B). The increase in molecular weight in CytoTDP fish compared to Control suggested post-translational modifications of Tardbp. Immunhistochemical stainings of CytoTDP as well as Western blot analysis with an antibody against phosphorylated TDP-43 at serine 409 and 410 did not reveal any positive signal. Additionally, we did not detect differences between CytoTDP and Control in a Western blot with an ubiquitin specific antibody suggesting other post-translational modifications induced by the ΔNLS-Tardbp mutation (SFigure 4A,B).

**Fig. 2:**
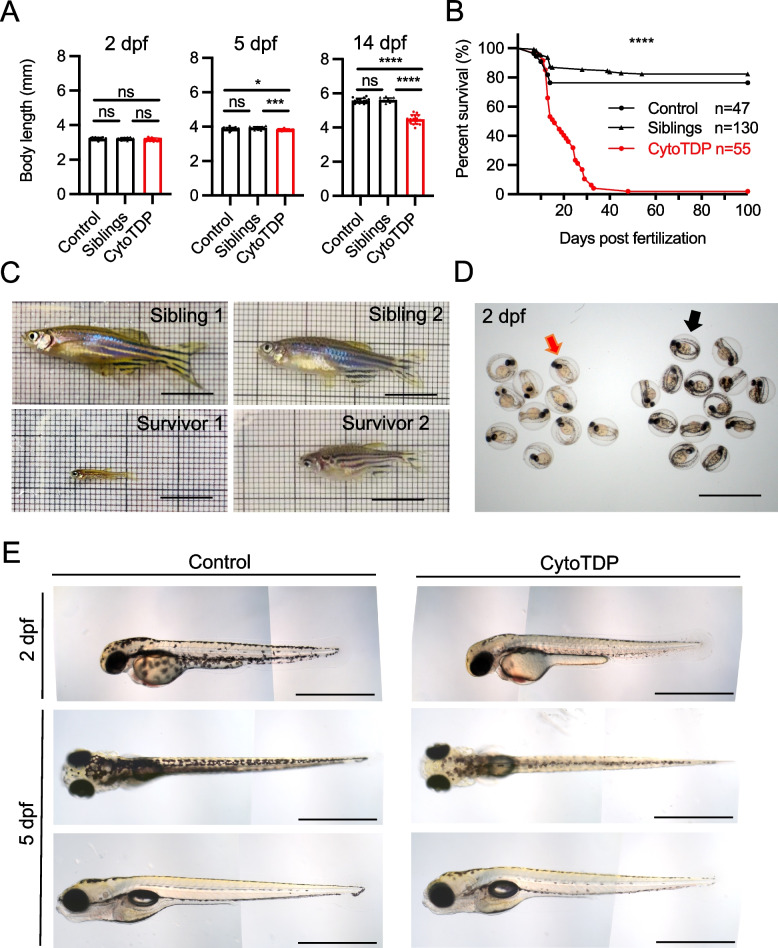
Morphological characterization of CytoTDP fish. **A** Quantification of body length of CytoTDP (red dots) and its siblings [Siblings (black arrow heads): *tardbp ΔNLS/* + *; tardbpl -/-*, Control (black dots): *tardbp* + */* + *; tardbpl -/-*] at 2 dpf, 5 dpf and 14 dpf. CytoTDP larvae have a similar body length at 2 dpf but are smaller at 5 dpf and even more severe at 14 dpf. 2 dpf ANOVA test [*p* = 0.4073, Unpaired T test: Control vs Siblings *p* > 0.9999, Control vs CytoTDP *p* = 0.2528, Siblings vs CytoTDP *p* = 0.2445]; 5 dpf ANOVA test [*p* = 0.0012, Unpaired T test: Control vs Siblings *p* = 0.0894, Control vs CytoTDP * p = 0.0414, Siblings vs CytoTDP *** p = 0.0002]; 14 dpf ANOVA test [*p* < 0.0001, Unpaired T test: Control vs Siblings *p* = 0.4227, Control vs CytoTDP **** p < 0.0001, Siblings vs CytoTDP **** p < 0.0001]; Control *n* = 16, Siblings *n* = 15, CytoTDP *n* = 16. Error bars indicates ± SD. **B** Kaplan Mayer blot of CytoTDP (*n* = 47) and its siblings [Control (*tardbp* + */* + *; tardbpl-/-*) (*n* = 55), Siblings (*tardbp ΔNLS/* + *; tardbpl-/-*) (*n* = 130)]. 94.55% of CytoTDP larvae die within one month, only 1.82% CytoTDP larvae survive longer than 3 months. Mantel-Cox test, *p* < 0.0001. **C** Pictures of 2 CytoTDP survivors, 5 months old (left panel) and 6 months old (right panel), respectively. Survivors are of smaller size compared to their siblings. Scale bar = 1 cm. **D** Petri dish with sorted 2 dpf CytoTDP larvae according to their pigmentation phenotype (red arrow) and their siblings (black arrow). Scale bar = 2 mm. **E** Hypopigmented CytoTDP embryos display strongly reduced pigmentation compared to their siblings at 2 dpf (lateral view, top panel). At 5 dpf the hypopigmentation pigmentation is still clearly visible from a dorsal view (middle panel) but is less pronounced from a lateral perspective (bottom panel). Scale bar = 1 mm

CytoTDP fish had a similar body length at 2 dpf compared to Siblings and Control but were smaller at 5 and 14 dpf (Fig. 2A). Most of the CytoTDP fish died within the first month after birth, only a few survived beyond one month of age (Fig. 2B), which were noticeably smaller than their siblings (Fig. 2C). Moreover, some CytoTDP fish had a distinct hypopigmentation phenotype that can be used as a specific morphological marker to identify these fish simply by eye without the need of a standard dissection microscope as early as 1.5 dpf (SFigure 5, Fig. 2D,E).

**Fig. 3:**
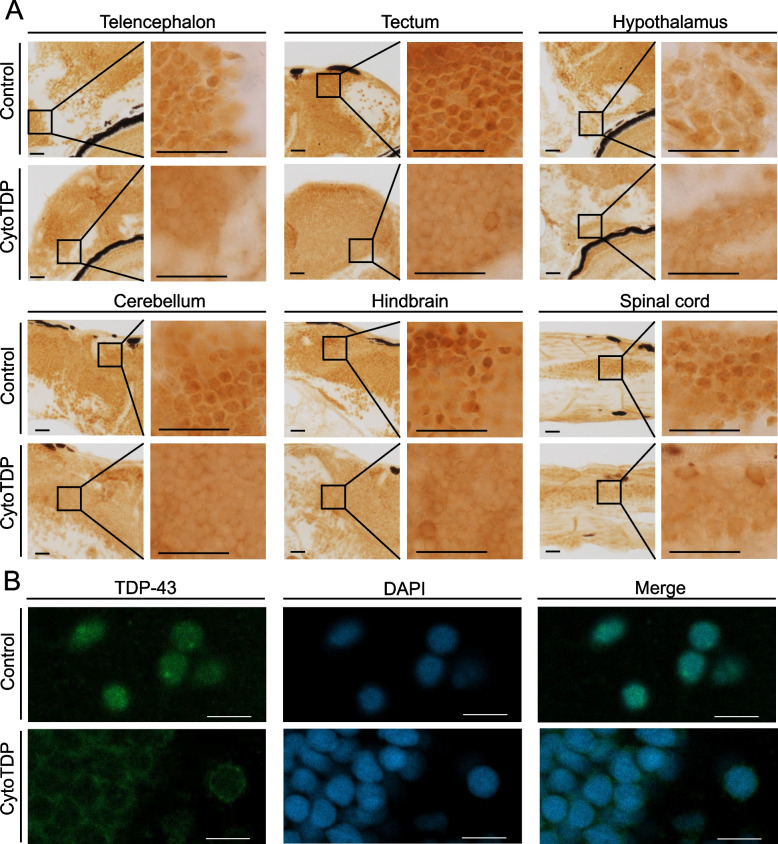
Tardbp localization in CytoTDP fish. **A** Immunohistochemical stainings of brain (including telencephalon, tectum, hypothalamus, cerebellum and hindbrain) and spinal cord paraffin sections of 5 dpf CytoTDP fish and Control fish show decreased level of Tardbp in the nucleus and increased cytoplasmic Tardbp expression in CytoTDP fish. Black boxes indicate magnified area. Scale bar = 100 μm. **B** Immunofluorescence stainings of 5 dpf CytoTDP fish and Control fish brain with Tardbp antibody (green) and DAPI (blue) highlighting the relocalization of Tardbp in CytoTDP line. Scale bar = 10 μm

To verify that the ΔNLS-Tardbp mutation induced Tardbp cytoplasmic mis-localization in CytoTDP, we stained brain and spinal cord paraffin sections for Tardbp and successfully confirmed increased cytoplasmic expression with concurrent decreased nuclear expression of ΔNLS-Tardbp in CytoTDP fish (Fig. 3A, B).

### Age dependent movement phenotypes

**Fig. 4:**
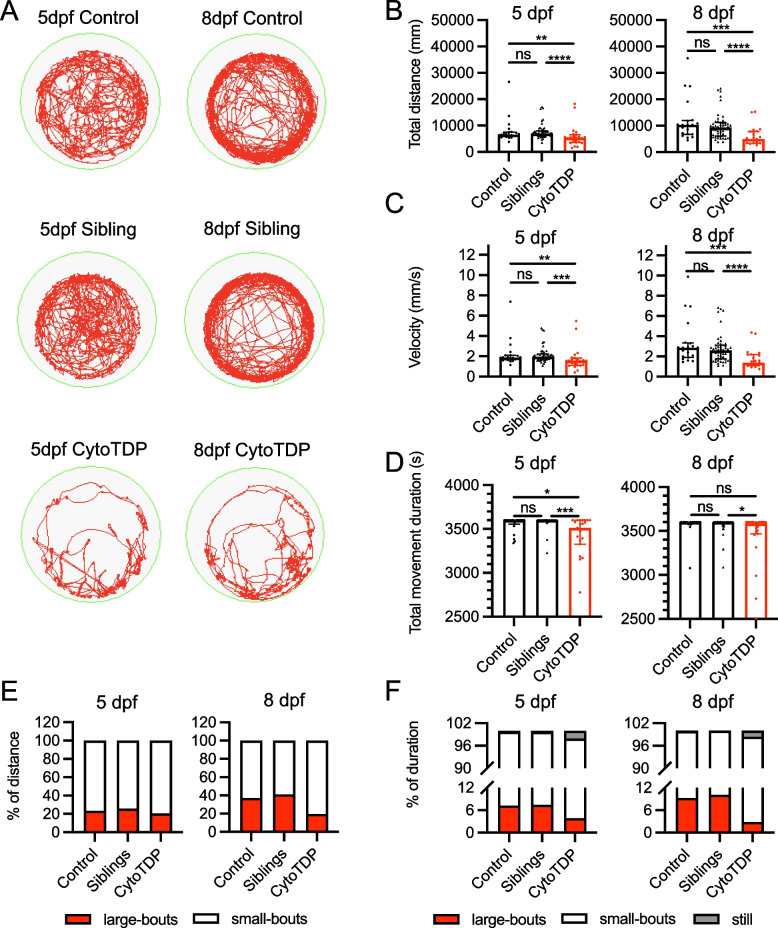
Age dependent movement phenotypes. **A** Representative locomotor activity path of 1 h video recordings (red path) of one larva of CytoTDP and its siblings [Siblings (*tardbp ΔNLS/* + *; tardbpl -/-*), Control (*tardbp* + */* + *; tardbpl -/-*)] at 5 dpf and 8 dpf. **B-D** Quantifications of total swimming distance, velocity and movement duration for CytoTDP (red dots) and its siblings [Siblings (black arrow heads): *tardbp ΔNLS/* + *; tardbpl -/-*, Control (black dots): *tardbp* + */* + *; tardbpl -/-*] at 5 dpf and 8 dpf. Kruskal–Wallis test was used for 3 groups comparisons and Mann–Whitney test was used for 2 groups comparisons. Error bars indicates ± interquartile range. Control *n* = 21, Siblings *n* = 50, CytoTDP *n* = 23. **B** Swimming distance [5 dpf (***p = 0.0003, Control vs Siblings *p* > 0.9999, Control vs CytoTDP **p = 0.0044, Siblings vs CytoTDP ****p < 0.0001); 8 dpf (****p < 0.0001, Control vs Siblings *p* > 0.9999, Control vs CytoTDP ***p = 0.0002, Siblings vs CytoTDP ****p < 0.0001)]; **C** Swimming velocity [5 dpf (***p = 0.0006, Control vs Siblings *p* = 0.3192, Control vs CytoTDP **p = 0.0052, Siblings vs CytoTDP **** p < 0.0001); 8 dpf (****p < 0.0001, Control vs Siblings *p* = 0.4565, Control vs CytoTDP ***p = 0.0001, Siblings vs CytoTDP **** p < 0.0001)] **D** Movement duration [5 dpf (**p = 0.0032, Control vs Siblings *p* = 0.5607, Control vs CytoTDP * p = 0.0403, Siblings vs CytoTDP ***p = 0.0004); 8 dpf (*p* = 0.1043, Control vs Siblings *p* = 0.9375, Control vs CytoTDP *p* = 0.0959, Siblings vs CytoTDP *p = 0.0427)]. **E** 100% stacked bar graph showing mean distribution of small-bouts (white bar) and large-bouts (red bar) swimming distance percentages for CytoTDP, Siblings and Control at 5 dpf and 8 dpf **F** 100% stacked bar graph showing mean distribution of durations of small-bouts (white bar), large-bouts (red bar) and still time with no movement (grey bar) percentages of Control, Siblings and CytoTDP at 5 dpf and 8 dpf. Control *n* = 21, Siblings *n* = 50, CytoTDP *n* = 23

ALS patients suffer from progressive muscle weakness and motor dysfunction [2]. An ALS-like phenotype in zebrafish should present with a progressive reduced swimming performance. We observed reduced touch-evoked escape responses already at 1.5 dpf in CytoTDP fish compared to their siblings (SVideo 1). Quantification of a successful escape response showed that around 85% of CytoTDP fish had reduced responses (SFigure 6A, B), suggesting motor neuron defects as early as 1.5 dpf. At the age of 5 dpf zebrafish larvae have used up all their yolk, are actively swimming and start to search food. We used video recording of spontaneous locomotor activity in 5 and 8 dpf old zebrafish larvae over a period of 1 h to assess the swimming performance. CytoTDP showed swimming deficits at both time points examined. They showed decreased total swimming distance (Fig. 4A,B) compared to their siblings already at 5 dpf indicative of a motor deficit in CytoTDP. The swimming velocity was also decreased in CytoTDP, they moved shorter distances at a lower speed compared to their siblings at 5 dpf (Fig. 4C). The decline in swimming distance and velocity became more pronounced at 8 dpf (Fig. 4B,C) and reflect the progressive nature of degeneration. Surprisingly, CytoTDP larvae only showed a slightly reduced duration of movement compared to their siblings at 5 dpf and 8 dpf (Fig. 4D).

Additionally, CytoTDP were unable to keep a proper posture and swam smaller distances once they initiated a swimming bout (short bursts of movement) (SVideo 2). To quantify these observations, we set a threshold according to moving distance per initiation of swimming bout to small-bouts (< 4 mm) versus large-bouts (≥ 4 mm). Strikingly, CytoTDP displayed significantly reduced amount and percentages of large swimming bouts compared to their siblings at 5 dpf. This movement deficit became more severe at 8 dpf (Fig. 4E, SFigure 7A-C). Additionally, CytoTDP exhibited a significant decrease in the amount and percentages of durations of large-bouts when compared to their siblings. This phenotype became more pronounced at 8 dpf (Fig. 4F, SFigure 7D-F), indicating that CytoTDP fish have a neuromuscular junction or muscular dysfunction.

### Progressive degeneration of neuromuscular junctions and muscle atrophy in CytoTDP

**Fig. 5:**
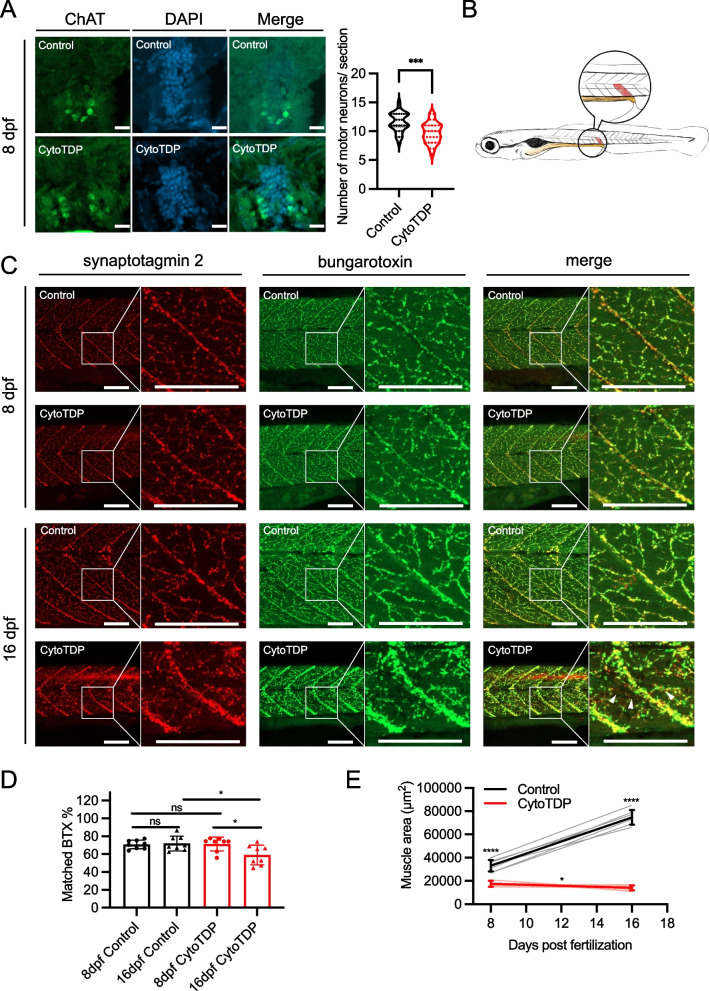
Reduction of motor neurons and NMJ degeneration in CytoTDP. **A** Representative images of ChAT stained 20 μm spinal cord sections from Control and CytoTDP at 8 dpf, scale bars = 20 μm. Quantification of ChAT-positive cells per 20 μm section of Control and CytoTDP at 8 dpf. Control vs CytoTDP *** p = 0.0001, unpaired T test, *n* = 30 sections for each group. **B** Schematic drawing of a 5 dpf old larva highlighting the last ventral half of the somite (red) before the end of the gut (yellow), which was used for quantifications. **C** Representative pictures of double whole-mount immunostainings for synaptotagmin 2 (presynaptic marker, red) and bungarotoxin (postsynaptic marker, green) highlighting the NMJ for CytoTDP and Control at 8 dpf and 16 dpf. CytoTDP and Control have similar NMJ structure at 8 dpf, while 16 dpf CytoTDP have misaligned presynaptic and postsynaptic staining indicative of a degenerated NMJ. Boxes indicate magnified area. Arrowheads in 16 dpf CytoTDP merge magnified image point to non-overlapping synaptotagmin 2 (red) and bungarotoxin (green) stainings. Scale bar = 150 µm. **D** Quantification of colocalized presynaptic and postsynaptic markers in the last ventral half of the somite before the end of the gut in CytoTDP and Control at 8 dpf and 16 dpf. Unpaired T test: 8 dpf Control vs 8 dpf CytoTDP *p* = 0.8819, 16 dpf Control vs 16 dpf CytoTDP * p = 0.0187, 8 dpf Control vs 16 dpf Control *p* = 0.7551, 8 dpf CytoTDP vs 16 dpf CytoTDP * p = 0.0215; each group *n* = 8; Error bars indicates ± SD. **E** Quantifications of musculature area of the last ventral half somite before the end of the gut in CytoTDP and Control fish at 8 dpf and 16 dpf. Unpaired T test: 8 dpf Control vs 8 dpf CytoTDP **** p < 0.0001, 16 dpf Control vs 16 dpf CytoTDP **** p < 0.0001, 8 dpf Control vs 16 dpf Control **** p < 0.0001, 8 dpf CytoTDP vs 16 dpf CytoTDP *p* = 0.0157; *n* = 7 larvae for each group. Error bars indicates ± SD

Muscle atrophy caused by loss of neuromuscular junctions (NMJs) and motor neurons is a hallmark of ALS [2, 28]. We therefore analyzed the motor neurons, NMJ and muscle atrophy. Quantifications of motor neuron numbers with an antibody against choline acetyltransferase (ChAT) revealed a significant reduction of ChAT-positive motor neurons in spinal cord of CytoTDP at 8 dpf (Fig. 5A). This finding, together with strong motor defects at 8 dpf, supports degeneration of motor neurons. We then quantified the colocalization of bungarotoxin (BTX) positive postsynapes with stainings of the presynaptic marker synaptotagmin 2 in the last ventral half of the somite before the end of the gut in CytoTDP and Control at 8 dpf and 16 dpf as a readout of NMJ integrity (Fig. 5B). CytoTDP showed a strong overlap of the presynaptic and postsynaptic marker with no abnormalities of the NMJ stainings compared to Control at 8 dpf. In contrast, at 16 dpf CytoTDP fish exhibited markedly reduced colocalization of presynaptic and postsynaptic markers indicative of NMJ defects (Fig. 5C,D). This indicates that the NMJs are properly formed at 8 dpf but degenerate over time as seen in ALS. To assess muscle size, we measured the area of the last somite dorsal of the end of the gut. In CytoTDP the somite was smaller compared to Control at 8 dpf and 16 dpf. Interestingly, the somite of CytoTDP at 16 dpf was not only smaller compared to Control, but also significantly smaller compared to CytoTDP at 8 dpf (Fig. 5E), indicative of progressive muscle atrophy.

**Fig. 6:**
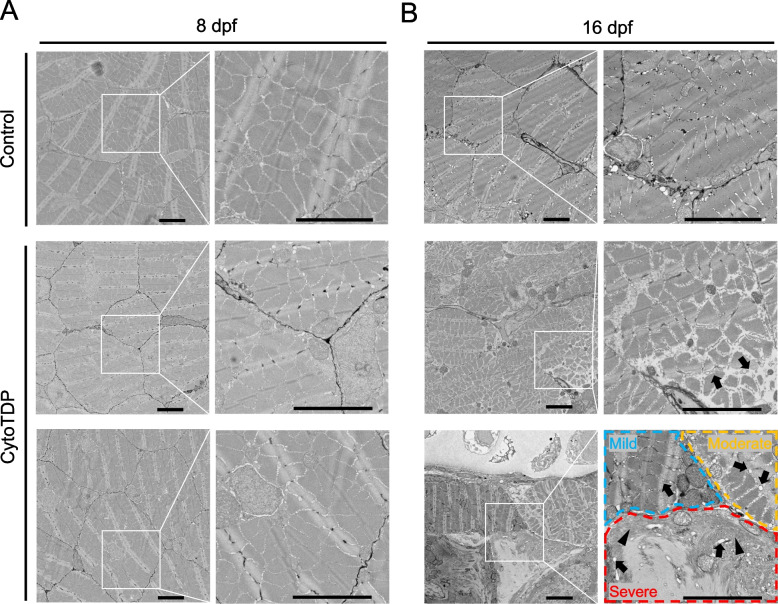
Mislocalization of endogenous TDP-43 causes muscle atrophy. **A** Representative electron microscopy (EM) images for CytoTDP and Control skeletal muscle at 8 dpf. CytoTDP have well-organized myofibrils and no obvious muscle atrophy at 8 dpf compared to Control. **B** Representative EM images for CytoTDP and Control skeletal muscle at 16 dpf. 16 dpf CytoTDP have a dilated sarcoplasmic reticulum (SR) (arrows) and significant muscle atrophy. Mildly affected muscle fiber (blue circled area) shows slightly dilated SR. Moderately affected muscle fiber (orange circled area) shows dilated SR. Severely affected muscle fiber (red circled area) shows dilated SR and severely misarranged cytoplasmic structures (arrowhead) (Scale bar = 5 μm)

To further investigate potential muscle atrophy in CytoTDP on an ultrastructural level, we performed scanning electron microscopy (SEM) on CytoTDP and Control fish at 8 dpf and 16 dpf. Consistent with the NMJ results, no signs of ultrastructural muscle atrophy were seen in CytoTDP at 8 dpf (Fig. 6A). Strikingly, CytoTDP showed varying degrees of muscle atrophy of the trunk musculature in individual fish in comparison to Control at 16 dpf. In mildly affected muscle fibers, we observed a minor dilation of the sarcoplasmic reticulum (SR). The dilation of SR was more pronounced in moderately affected muscle fibers. In severely affected muscle fibers, the cytoplasmic muscle cells structures were completely disorganized (Fig. 6B).

### Microglia proliferation and activation in the hypothalamus of CytoTDP

**Fig. 7:**
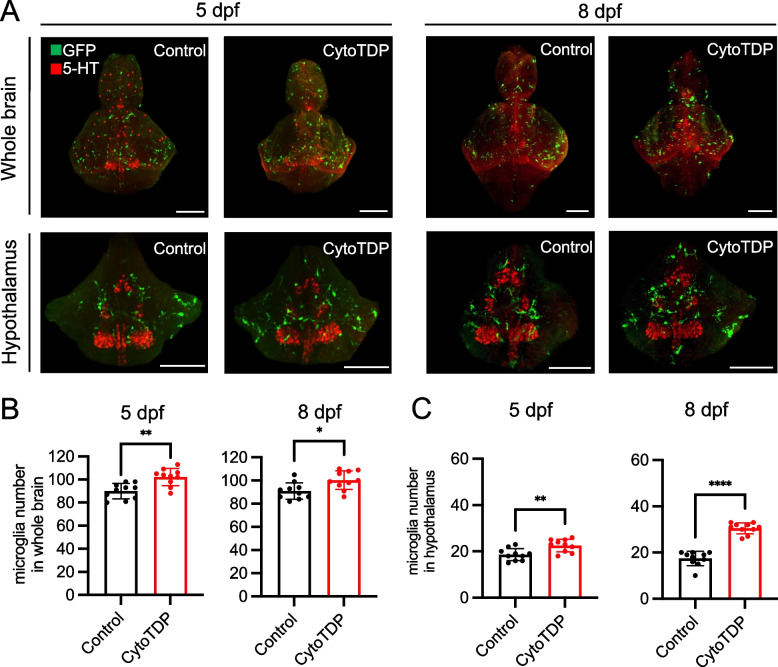
Microglia proliferation in the hypothalamus of CytoTDP. **A** Representative dorsal views of segmented whole-brain (top panel) and hypothalamus (lower panel) images using tissue clearing and whole-mount immunostaining of microglia at 5 dpf and 8 dpf. Co-staining against 5-hydroxytryptamine (5-HT) was used as a counterstain for the hypothalamus in zebrafish [38]. Confocal scans of mpeg1.1-eGFP positive microglia (green) and 5-HT (red) CytoTDP and Control larvae. Scale bars = 100 μm. **B** Quantification of microglia cell number in whole-brain of Control versus CytoTDP larvae at 5 dpf and 8 dpf, 5 dpf Control vs 5 dpf CytoTDP ** p = 0.0012, 8 dpf Control vs 8 dpf * p = 0.0134, *n* = 10 larvae for each group, unpaired T test, Error bars indicates ± SD. **C** Quantification of microglia cell number in the hypothalamus of Control versus CytoTDP larvae at 5 dpf and 8 dpf, 5 dpf Control vs 5 dpf CytoTDP ** p = 0.0041, 8 dpf Control vs 8 dpf CytoTDP **** p < 0.0001, unpaired T test, *n* = 10 larvae for each group, Error bars indicates ± SD

**Fig. 8:**
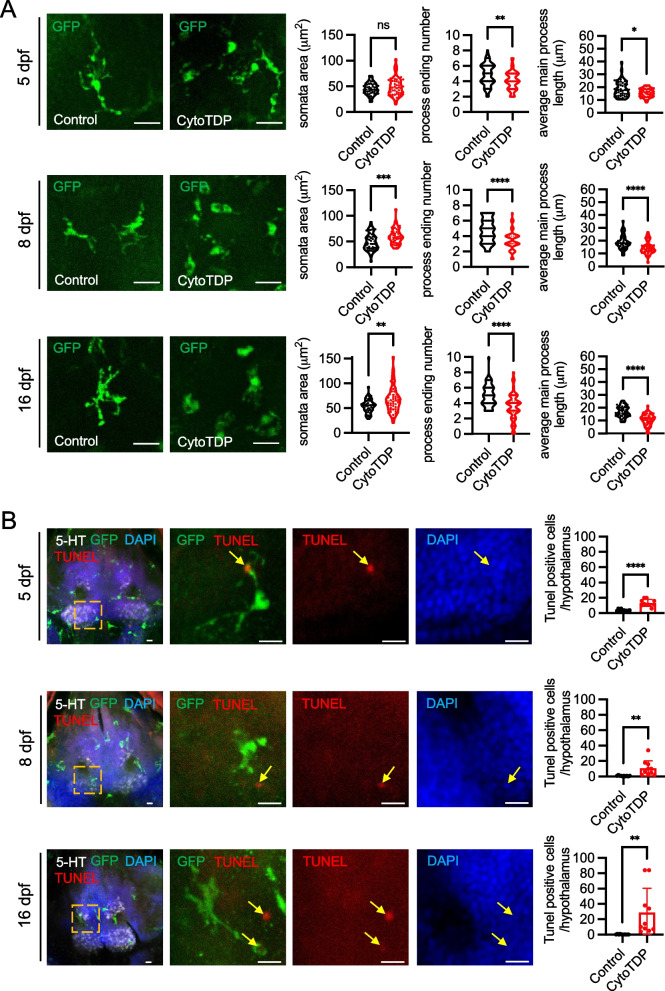
Microglia activation in the hypothalamus of CytoTDP. **A** Representative images of mpeg1.1-eGFP positive microglia in hypothalamus from Control and CytoTDP at 5 dpf, 8 dpf and 16 dpf. Scale bars = 20 μm. Violin plots show the area of somata, number of main processes, and average main process length of mpeg1.1-eGFP positive microglia in CytoTDP and Control at 5 dpf, 8 dpf and 16 dpf. 5 dpf (somata area *p* = 0.2165, number of main processes ** p = 0.0068, average main process length * p = 0.0308), 8 dpf (somata area *** p = 0.0008, number of main processes **** p < 0.0001, average main process length **** p < 0.0001), 16 dpf (somata area ** p = 0.003, number of main processes **** p < 0.0001, average main process length **** p < 0.0001), *n* = 50 cells for each group, unpaired T test. Each point represents one cell. **B** Representative maximum intensity projections of confocal images of mpeg1.1-eGFP CytoTDP fish after whole-mount immunostaining with tissue clearing and quantifications of TUNEL positive cells in the hypothalamus of CytoTDP fish and Control fish at 5 dpf, 8 dpf and 16dpf. 5-HT, mpeg1.1-eGFP positive microglia, TUNEL and DAPI signals are in gray, green, red and blue, respectively. Colocalization of TUNEL apoptotic DNA fragments, DAPI stained nuclei and microglia processes (yellow arrows) are evident in CytoTDP zebrafish through all the checked time points. Scale bars = 10 μm. 5 dpf Control vs 5 dpf CytoTDP **** p < 0.0001, 8 dpf Control vs 8 dpf CytoTDP ** p = 0.0061, 16 dpf Control vs 16 dpf CytoTDP ** p = 0.0099, unpaired T test, *n* = 10 larvae for each group, Error bars indicates ± SD

We next investigated brain pathology in CytoTDP fish. As the most abundant phagocytes in the central nervous system (CNS), microglia form a network that extends across the CNS and can sense changes in the environment [29, 30]. Microglia are also responsible for elimination of dead cells [31]. Thus, CNS pathologies can often be first identified by activation of microglia. To assess potential affected brain areas, we used the microglia reporter line Tg(mpeg1.1: EGFP-CAAX) [32] to label microglia in the CytoTDP background. Potentially affected brain areas at early stage in both CytoTDP versus Control zebrafish were analyzed by using whole-mount immunostaining and tissue clearing (SVideo 3). We compared the microglial abundance at 5 dpf and 8 dpf in whole-brain and found a significant increase of total microglia in CytoTDP compared to Control (Fig. 7A,B). Since CytoTDP fish were significantly smaller and metabolic dysfunction has been demonstrated as a prevalent primary characteristic in the initial phases of ALS [33–36], we investigated if the hypothalamus as a metabolic regulatory center might be selectively affected in CytoTDP fish [37]. Strikingly, microglia accumulated significantly in the CytoTDP hypothalamus compared to Control at 5 dpf, with an even more pronounced accumulation at 8 dpf (Fig. 7A,C). We also observed morphological signatures consistent with microglia activation such as larger somata, shorter and reduced number of processes in CytoTDP (Fig. 8A). To identify potential causes of microglia activation, we analyzed cell death by quantification of TUNEL positive cells (Fig. 8B). We found significantly increased numbers of dying cells in all stages analyzed in the hypothalamus of CytoTDP fish compared to Control fish. These findings indicate that the hypothalamus is a significantly affected brain region by Tardbp mis-localization in this model.

### CytoTDP affects key metabolic processes

**Fig. 9:**
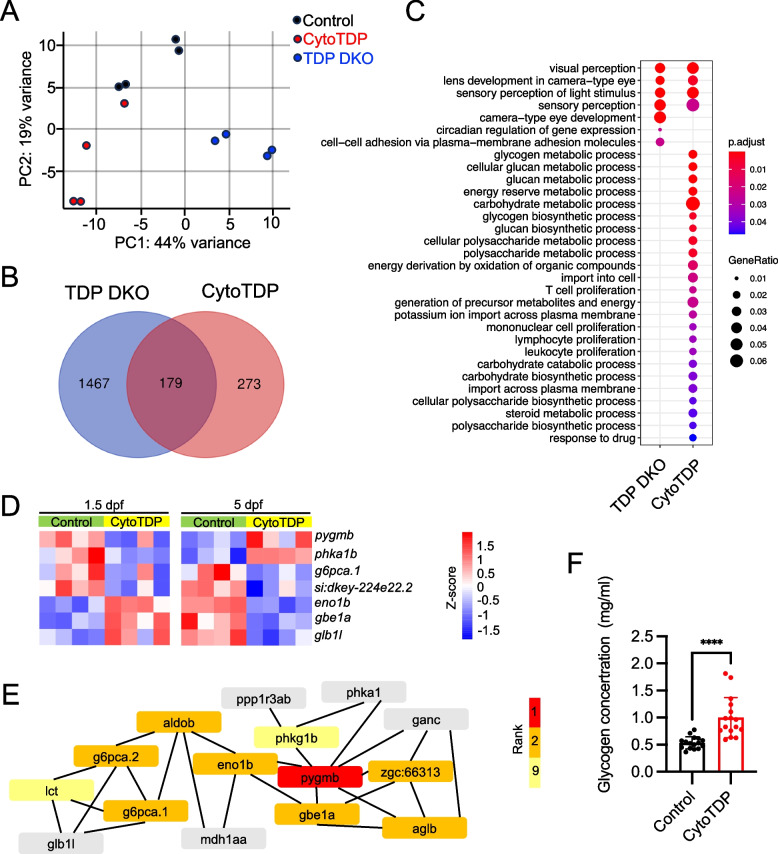
CytoTDP affects key metabolic processes. **A** Principal component analysis (PCA) of RNA sequencing results showed a strong separation of genotypes among the three groups of zebrafish (TDP DKO, CytoTDP and Control). **B** Venn diagram representation of the number of differentially expressed genes in TDP DKO (blue) and CytoTDP (red) compared to Control. **C** Gene ontology analysis of the biological process of genes differentially expressed of TDP DKO and CytoTDP compared to Control, respectively. **D** Heatmap of significantly mis-regulated genes in carbohydrate metabolic pathways in 1.5 dpf and 5 dpf CytoTDP fish. **E** Hub genes were identified through Cytoscape, with the color intensity representing the gene's ranking. **F** Quantification of glycogen concentrations revealed that the glycogen concentrations in 5 dpf CytoTDP embryos (*n* = 16) were 1.87 fold higher than in Control (*n* = 16) (****p < 0.0001, Unpaired T test)

To perform an unbiased analysis of the early pathological molecular changes of TDP-43 mis-localization in disease, we performed RNA sequencing on zebrafish at 1.5 dpf, the earliest stage CytoTDP and TDP DKO (*tardbp -/- ; tardbpl -/-)* mutants can be identified morphologically by a pigmentation and hypoperfusion phenotype, respectively (STable 1). Principal component analysis (PCA) showed a strong separation of genotypes among the three groups of zebrafish analyzed: CytoTDP, TDP DKO and Control (Fig. 9A). Importantly, this analysis showed that the effects of Tardbp mis-localization are not identical to the TDP DKO enabeling the identification of cytoplasmic gain of function targets of Tardbp. Venn analysis revealed that CytoTDP and TDP DKO have 179 overlapping dysregulated genes, 1467 genes are exclusively dysregulated in TDP DKO and 273 genes are specifically dysregulated in CytoTDP (Fig. 9B). Gene ontology (GO) analysis identified that enrichment of overlapping genes mainly concentrated in visual perception, lens development in camera-type eye, sensory perception of light stimulus and sensory perception, which identifies these mis-regulated pathways as a cause of Tardbp loss of nuclear function (Fig. 9C).

Interestingly, GO analysis revealed that many metabolic pathways were only changed in CytoTDP, suggesting that Tardbp mis-localization rather than Tardbp nuclear deficiency can cause metabolic dysfunction. Most strikingly, many pathways involving glycogen and carbohydrate metabolism were affected (e.g. glycogen metabolic process, glycogen biosynthetic process, carbohydrate metabolic process), as well as immune cell related processes (e.g. T-cell proliferation, leukocyte proliferation, and lymphocyte proliferation), and sterol metabolic processes (Fig. 9C). In all CytoTDP’s mis-regulated pathways, carbohydrate metabolic processes had the highest number of mis-regulated genes. These carbohydrate metabolism associated genes were subsequently analyzed at a later timepoint (5 dpf). In line with our findings at 1.5 dpf, we identified mis-regulation of many genes in carbohydrate metabolic pathways in 5 dpf CytoTDP (Fig. 9D). Mis-regulation of metabolic genes in the opposite direction at 5 dpf compared to 1.5 dpf might be mediated by counter-regulatory mechanisms through disturbed glycogen homeostasis. To evaluate which genes play a central role at early stage, we identified hub genes, which are characterized by a high connectivity within gene networks [26] (Fig. 9E). The top ranked gene in this analysis was muscle glycogen phosphorylase b (*pygmb*), which showed a decrease of 41% in 1.5 dpf CytoTDP fish. *pygmb* is a key enzyme in glycogenolysis to release glucose from glycogen. It has been previously shown that *pygmb* can affect glycogen levels in zebrafish embryos [39]. To address the consequences of carbohydrate metabolic dysfunction in CytoTDP, we measured the glycogen levels and found that glycogen levels were increased in CytoTDP compared to Control (Fig. 9F), confirming our findings that Tardbp mis-localization significantly altered carbohydrate processing in CytoTDP zebrafish.

## Discussion

An animal model that combines both features of nuclear clearing of TDP-43 and endogenously increased cytoplasmic TDP-43 is an important but still missing part in ALS research. Here, we generated a novel animal model, called CytoTDP, that re-locates endogenous Tardbp from the nucleus to the cytoplasm to identify mis-regulated pathways that are affected in early ALS. We show that nuclear clearing of TDP-43 and endogenous accumulation of cytoplasmic TDP-43 lead to early phenotypes, including rapidly progressing motor defects, neuromuscular junction disruptions, muscle atrophy and metabolic dysfunction in CytoTDP. Whole-brain microglia imaging revealed microglia proliferation and activation, as well as increased cell death in the hypothalamus prior food intake at 5 dpf. In addition, we show that CytoTDP, in contrast to TDP DKO fish, have early onset mis-regulated metabolic carbohydrate pathways reminiscent of early stage of ALS/FTLD. Thus, our CytoTDP animal model can provide a valuable tool for the understanding of pathological processes and the development of new metabolism targeting treatments in early stage ALS.

In 2006, TDP-43 nuclear clearing and endogenous cytoplasmic inclusions were identified to be a hallmark in ALS/FTLD [4]. We still do not have animal models to mimic this TDP-43 pathology that happens in more than 90% ALS cases [10], which dramatically slows understanding of TDP-43 function in ALS disease. While there is increasing evidence for a loss of TDP-43 function contribution, such as *STMN2* and *UNC13A* mis-splicing [40–42], the contribution of cytoplasmic TDP-43 gain of function has been less clearly demonstrated. To study the gain of cytoplasmic TDP-43 function, transgenic mice (rNLS8) that express human TDP-43-ΔNLS (hTDP-43-ΔNLS) in the brain and spinal cord were generated [43]. These mice have > tenfold higher expression level than endogenous TDP-43, which is different from human ALS disease cases. Significantly lower levels of human WT TDP-43 overexpression (3.8 fold) in transgenic mice already lead to mortality at ~ 6 months of age indicating that high exogenous expression of TDP-43 leads to toxicity [13]. To circumvent this problem, several new knockin TDP-43 animal models have been generated to study endogenous mutant TDP-43. However, these models only have subtle motor ALS-like phenotype with a very late onset, which makes them less suitable for drug discovery [9, 44, 45]. Thus, new knockin TDP-43 animal models that combine both features of nuclear clearing of TDP-43 and increased cytoplasmic TDP-43, as in the zebrafish model described here, are needed to accurately reproduce the intrinsic molecular signature of early disease pathology.

Our newly generated CytoTDP zebrafish successfully recapitulates ALS in multiple important clinical aspects at a very early stage, including progressive motor defects, neuromuscular junction degeneration and muscle atrophy. In the CytoTDP line, normal NMJ structures were formed first, but then degenerate, which better mimics the neurodegenerative features of ALS than other reported zebrafish TDP-43 models [46]. In addition, motor defects in CytoTDP started before the morphological neuromuscular junction degeneration, indicating the primary motor defects are caused by ALS early stage neuron dysfunction. Motor defects in CytoTDP fish start already at 1.5 dpf, which is a great advantage over TDP-43 mutation KI zebrafish and rodent models, which only show phenotypes at later stages [10, 45, 47].

We use whole-mount immunolabeling and whole-brain microglia imaging in an ALS animal model for the first time, allowing systemic analysis of microglia morphology in the brain. Cleared brain imaging revealed increased number and activation of microglia in CytoTDP, which is consistent with findings in human ALS and other animal models [48–51], with an pronounced microglial activation and cell death in the hypothalamus, a central metabolic regulatory center [37, 52]. In patients with ALS, metabolic dysfunction has been shown to be a common primary feature of early ALS prior to motor defects. A key question yet to be resolved in humans is to rule out the impact of decreased food intake caused by muscle weakness, problems to swallow or potential depression on metabolic dysfunction [33, 53]. Interestingly, the activation of hypothalamic microglia occurs before the onset of food intake in zebrafish embryos at 5 dpf and becoming more severe over time, arguing for a direct effect on the hypothalamus. Importantly, atrophy as well as TDP-43 pathology has been observed in the hypothalamus of ALS patients [54–57] arguing for hypothalamic involvement to disease ethiology. In ALS patients, weight loss triggered by decreased food intake, including reduced appetite and dysphagia or metabolic disturbances are prominent features, and has been proven to be directly associated with poor ALS prognosis [35, 53, 58–61]. However, in previous human ALS patient and ALS animal model studies, the temporal relationship between decreased food intake, metabolic disturbances and weight loss in ALS patients remained unclear. By utilizing the CytoTDP line and combining whole-mount immunostaining and tissue clearing techniques, we have identified that microglia activation in the metabolic regulatory center in the CytoTDP line occur before the onset of food intake. This provides new insights and directions for future development of ALS drugs that can alleviate metabolic dysfunction at an early stage, thereby counteracting or minimizing disease progression.

In the past two decades, researchers have been trying to determine whether TDP-43-mediated neurodegenerative diseases are caused by the loss of nuclear function or gain of cytoplasmic toxic function of endogenous TDP-43. However, progress in this area has been slow due to embryonic lethality in TDP-43 knockout rodent models and the lack of animal models with mis-localized endogenous TDP-43 [62–64]. In this study, we made significant advances in investigating this question by analyzing the transcriptomes of our newly established CytoTDP model and our previously reported TDP DKO zebrafish model [14]. We found that compared to the TDP DKO model, CytoTDP showed dysregulation of central metabolic pathways, including glygogen storage and degradation, consistent with the muscle degeneration and activation of microglia in the hypothalamus. Through analysis of hub genes, we identified the important role of *pygmb* in the metabolic dysfunction of the CytoTDP model. Zebrafish have 2 orthologous genes encoding muscle isoform of glycogen phosphorylase: *pygma* and *pygmb*, both show great similarity to human muscle form of glycogen phosphorylase (*PYGM*). CytoTDP showed higher levels of glycogen, consistent with alterations in important enzymes in glycogen metabolic pathways [39]. Importantly, we examined differentially expressed genes in human C9orf72 ALS and found that the *PYGM* gene was also downregulated in the motor cortex of patients [50]. Other studies have shown that the pathology of TDP-43 in ALS occurs first in the motor cortex [65], suggesting that the downregulation of *PYGM* in the motor cortex of ALS patients may be crucial for understanding the early mechanisms of ALS. PYGM is a key enzyme in glycogen metabolism catalyzing glycogen cleavage into glucose-1-phosphate, expressed not only in skeletal and cardiac muscles [66], but also at considerable concentrations in the brain, mainly in astrocytes [67–70]. The functional consequences of the downregulation of *PYGM* in the motor cortex of ALS patients are still unclear. However, another study using astrocytes differentiated from C9orf72 ALS patients have shown that the downregulation of *PYGM* is associated with mitochondrial energy loss and impaired glycolysis [71]. Therefore, our findings support the hypothesis that the downregulation of *PYGM* in the motor cortex of ALS patients affects glycogen metabolism and energy homeostasis. It will be important to further explore the role of cellular energy metabolism on neurons and glial cells in future studies.

Consistent with our findings, increased glycogen storage in the central nervous system has been observed in ALS patients and ALS animal models [72]. Furthermore, some FDG-PET studies in ALS patients have linked reduced glucose uptake and phosphorylation with disease severity and early diagnosis of ALS [73, 74]. Despite these clear correlations in patients and in animal models, it remains unclear how these metabolic changes ultimately lead to neurodegeneration. Some clinical studies have shown that nutritional interventions in ALS patients can prolong survival or have a disease-modulating effect. However, these studies have limitations such as small group sizes, higher dropout rates than typical drug trials, difficulties in getting good nutritional balance, and modest benefits [75–78]. The CytoTDP ALS model can be used in the future to define the molecular mechanisms how TDP-43 interferes with glycogen metabolism and how these changes affect neurodegeneration.

## Conclusion

Overall, our CytoTDP model exhibits both the endogenous TDP-43 loss of nuclear function and cytoplasmic toxic gain of function, along with key features of ALS/FTLD. Importantly, our results indicate disturbances of metabolic regulatory center start prior the influence of reduced food intake in ALS patients. By comparing the CytoTDP model with the TDP DKO model, we discovered that the cytoplasmic toxicity of TDP-43, rather than the loss of nuclear function, contributes to early stage metabolic dysfunction. Therefore, our model will foster the understanding of the early mechanisms underlying ALS/FTLD.

## Supplementary Information

Supplementary Material 1: STable 1. CytoTDP RNA sequencing results of 1.5 dpf old larvae.

Supplementary Material 2: SFigure 1. Generation of zebrafish ΔNLS-Tardbp mutation. Schematic drawing representation of CRISPR/Cas9 genome editing strategy to generate the ΔNLS-Tardbp line and sequence reads confirming successful genome editing of NLS1 in ΔNLS-Tardbp homozygous fish.

Supplementary Material 3: SFigure 2. *tardbp ΔNLS/ΔNLS* fish have no obvious phenotype. (A) Lateral view of *tardbp* + */* + and *tardbp ΔNLS/ΔNLS* fish at 2 dpf, 5 dpf and 5 months of age. (B) Percent survival of *tardbp* + */* + (*n* = 39), *tardbp ΔNLS/* + (*n* = 102) and *tardbp ΔNLS/ΔNLS* (*n* = 45) animals over 100 dpf show no significant difference, Mantel-Cox test, *p* = 0.2666.

Supplementary Material 4: SFigure 3. ΔNLS-Tardbp can be detected by 2 independent antibodies. (A) Schematic drawing shows different binding sites for the 4A12 and 30G5 Tardbp antibody. (B) Western blot analysis with Tardbp antibodies 4A12 and 30G5 reveal Tardbp levels in CytoTDP and Control embryos from 2 to 5 dpf. Asterics mark unspecific bands. α-tubulin serves as a loading control.

Supplementary Material 5: SFigure 4. PhosphoTDP-43 staining, immunoblot and ubiquitin immunoblot show no obvious difference. (A) Immunohistochemical phosphoTDP-43 stainings of whole brain (including tectum, hypothalamus, cerebellum and hindbrain) and spinal cord paraffin sections of 5 dpf CytoTDP fish and Control fish show no differences between Control and CytoTDP fish. Scale bar = 100 μm. (B) Western blot analysis with a phospho TDP-43 antibody and an ubiquitin antibody for CytoTDP and Control larvae at 5 dpf. α -tubulin serves as a loading control.

Supplementary Material 6: SFigure 5. Decreased pigmentation phenotype in 1.5 dpf CytoTDP fish. Petri dish with sorted 1.5 dpf CytoTDP larvae according to their pigmentation phenotype (red arrow) and their siblings (black arrow). Scale bar = 2 mm.

Supplementary Material 7: SFigure 6. 1.5 dpf CytoTDP fish have reduced touch-evoked response. (A) Representative snapshot images of 1.5 dpf Control and zebrafish during touch-evoked response assay. (B) Quantification of normal response percentage for CytoTDP (red dots) and its siblings [Siblings (black arrow heads): *tardbp ΔNLS/* + *; tardbpl -/-*, Control (black dots): *tardbp* + */* + *; tardbpl -/-*] at 1.5 dpf. Kruskal–Wallis test was used for 3 group comparisons and Mann–Whitney test was used for 2 group comparisons. Error bars indicates ± interquartile range. Control *n* = 8, Siblings *n* = 15, CytoTDP *n* = 7. **** p < 0.0001, Control vs Siblings *p* = 0.2128, Control vs CytoTDP **** p < 0.0001, Siblings vs CytoTDP **** p < 0.0001.

Supplementary Material 8: SFigure 7. Detailed CytoTDP behaviour data. (A-B) Quantifications of small-bouts and large-bouts distance pencentage for CytoTDP (red dots) and its siblings [Siblings (black arrow heads): *tardbp ΔNLS/* + *; tardbpl -/-*, Control (black dots): *tardbp* + */* + *; tardbpl -/-*] at 5 dpf and 8 dpf. Kruskal–Wallis test was used for 3 group comparisons and Mann–Whitney test was used for 2 group comparisons. Error bars indicates ± interquartile range. Control *n* = 21, Siblings *n* = 50, CytoTDP *n* = 23. (A) Small-bouts distance pencentage [5 dpf (**p = 0.0015, Control vs Siblings *p* > 0.9999, Control vs CytoTDP * p = 0.0324, Siblings vs CytoTDP ** p = 0.0012); 8 dpf (****p < 0.0001, Control vs Siblings *p* > 0.9999, Control vs CytoTDP ** p = 0.0023, Siblings vs CytoTDP **** p < 0.0001)]; (B) Large-bouts distance percentage for CytoTDP and its siblings at 5 dpf and 8 dpf. [5 dpf (**p = 0.0015, Control vs Siblings *p* > 0.99, Control vs CytoTDP * p = 0.0375, Siblings vs CytoTDP ** p = 0.0011); 8 dpf (****p < 0.0001, Control vs Siblings *p* > 0.9999, Control vs CytoTDP ** p = 0.0023, Siblings vs CytoTDP **** p < 0.0001)]; (C) Stacked bar graph showing mean distribution of small-bouts distance (white bar) and large-bouts distance (red bar) for CytoTDP and its siblings at 5 dpf and 8 dpf. (D-E) Quantifications of small-bouts and large-bouts duration percentage for CytoTDP and its siblings at 5 dpf and 8 dpf. Kruskal–Wallis test was used for 3 group comparisons and Mann–Whitney test was used for 2 group comparisons. Error bars indicates ± interquartile range. Control *n* = 21, Siblings *n* = 50, CytoTDP *n* = 23. (D) Small-bouts duration pencentage [5 dpf (**p = 0.0015, Control vs Siblings *p* > 0.99, Control vs CytoTDP * p = 0.0324, Siblings vs CytoTDP ** p = 0.0012); 8 dpf (****p < 0.0001, Control vs Siblings *p* > 0.9999, Control vs CytoTDP ** p = 0.0023, Siblings vs CytoTDP **** p < 0.0001)] (E) Large-bouts duration percentage for CytoTDP and its siblings at 5 dpf and 8 dpf. [5 dpf (**p = 0.0015, Control vs Siblings *p* > 0.9999, Control vs CytoTDP * p = 0.0375, Siblings vs CytoTDP ** p = 0.0011); 8 dpf (****p < 0.0001, Control vs Siblings *p* > 0.9999, Control vs CytoTDP ** p = 0.0023, Siblings vs CytoTDP **** p < 0.0001)] (F) Stacked bar graph showing mean distribution of small-bouts (white bar) duration, large-bouts (red bar) duration and still duration (grey bar) for CytoTDP and its siblings at 5 dpf and 8 dpf.

Supplementary Material 9: SVideo 1. Mis-localization of endogenous TDP-43 affects behavior of 1.5 day old zebrafish larvae.

Supplementary Material 10: SVideo 2. CytoTDP (marked with red label) shows more small-distance swimming activity and abnormal swimming balance at 8 dpf compared to a control sibling.

Supplementary Material 11: SVideo 3. 3D reconstruction and rendering of whole zebrafish scans with tissue clearing and confocal microscopy.

## Data Availability

Raw data is included in the supplementary material or will be provided by the authors upon reasonable request.

## Abbreviations

5-HT: 5-Hydroxytryptamine
ALS: Amyotrophic lateral sclerosis
BTX: Bungarotoxin
ChAT: Choline acetyltransferase
CNS: Central nervous system
dpf: Days post fertilization
FTLD: Frontotemporal lobar degeneration
GO: Gene ontology
LCD: Low-complexity domain
nAChR: Nicotinic acetylcholine receptor
NLS: Nuclear localization sequence
NMJ: Neuromuscular junctions
PBS: Phosphate-buffered saline
PCA: Principal component analysis
PFA: Paraformaldehyde
RFLP: Restriction length polymorphism
RRM: RNA recognition motif
SEM: Scanning electron microscopy
SR: Sarcoplasmic reticulum
TARDBP: Transactive response DNA binding protein
Tardbpl: Tar DNA binding protein of 43 kDa-like
Tardbpl_tv1: *tardbpl* Transcript variant 1
TDP-43: TAR DNA-binding protein of 43 kDa
