# Supplementary figures and images for: Mis-localization of endogenous TDP-43 leads to ALS-like early-stage metabolic dysfunction and progressive motor deficits
Source: Mol Neurodegener. 2024 Jun 20;19:50. doi: 10.1186/s13024-024-00735-7 (PMC11188230; doi:10.1186/s13024-024-00735-7)

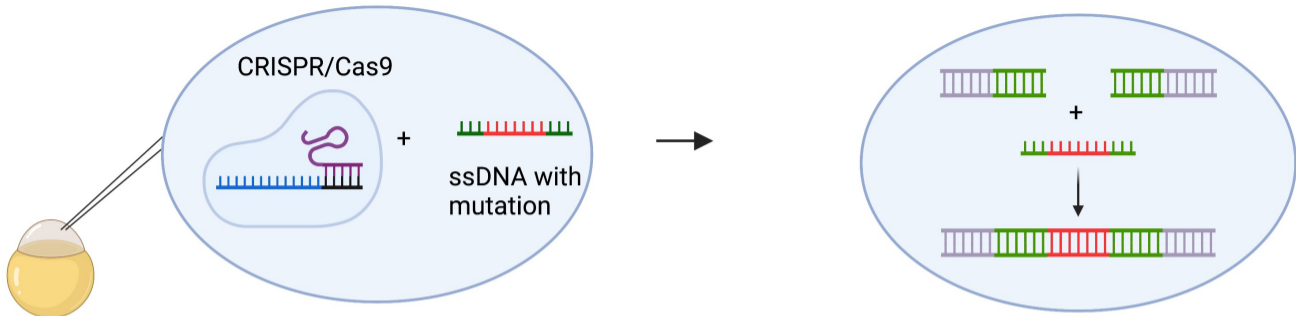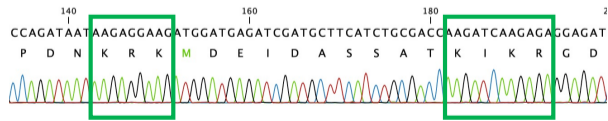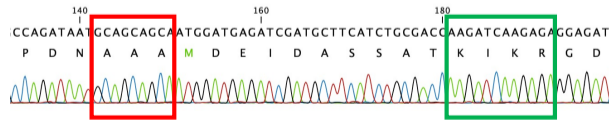

Supplement: Supplementary file 2 — Supplementary Material 2: SFigure 1. Generation of zebrafish ΔNLS-Tardbp mutation. Schematic drawing representation of CRISPR/Cas9 genome editing strategy to generate the ΔNLS-Tardbp line and sequence reads confirming successful genome editing of NLS1 in ΔNLS-Tardbp homozygous fish. [file 13024_2024_735_MOESM2_ESM.pdf]

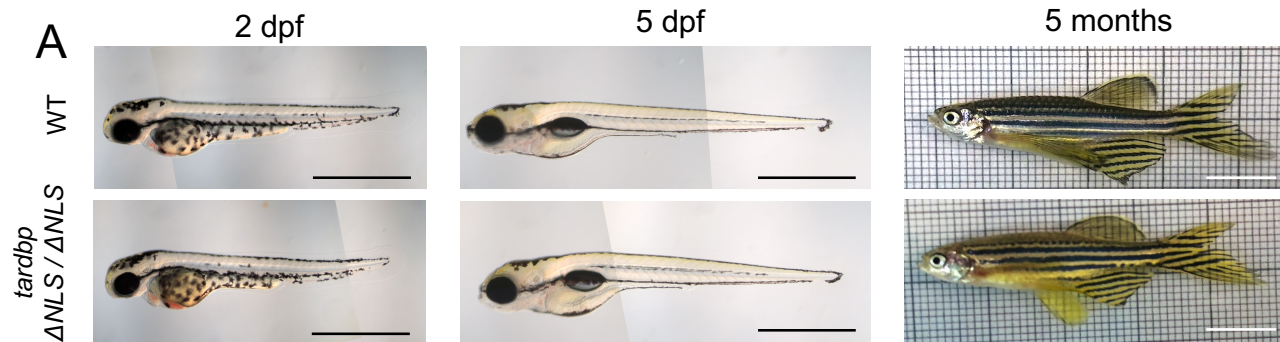

**B**

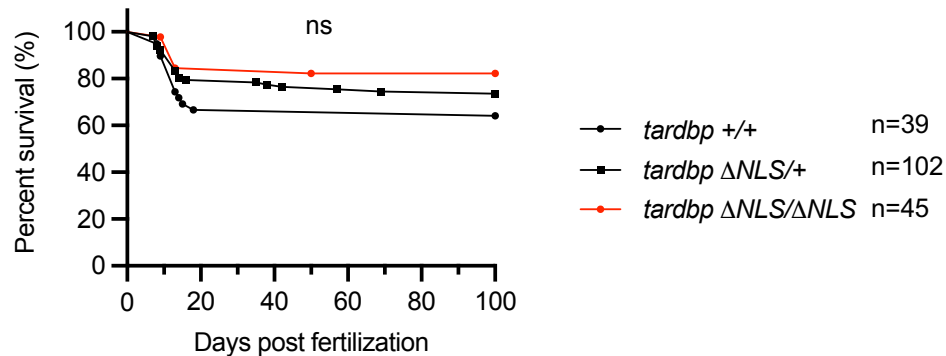

Supplement: Supplementary file 3 — Supplementary Material 3: SFigure 2. tardbp ΔNLS/ΔNLS fish have no obvious phenotype. (A) Lateral view of tardbp + / + and tardbp ΔNLS/ΔNLS fish at 2 dpf, 5 dpf and 5 months of age. (B) Percent survival of tardbp + / + (n = 39), tardbp ΔNLS/ + (n = 102) and tardbp ΔNLS/ΔNLS (n = 45) animals over 100 dpf show no significant difference, Mantel-Cox test, p = 0.2666. [file 13024_2024_735_MOESM3_ESM.pdf]

# A

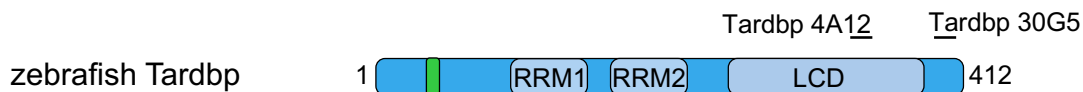

# B

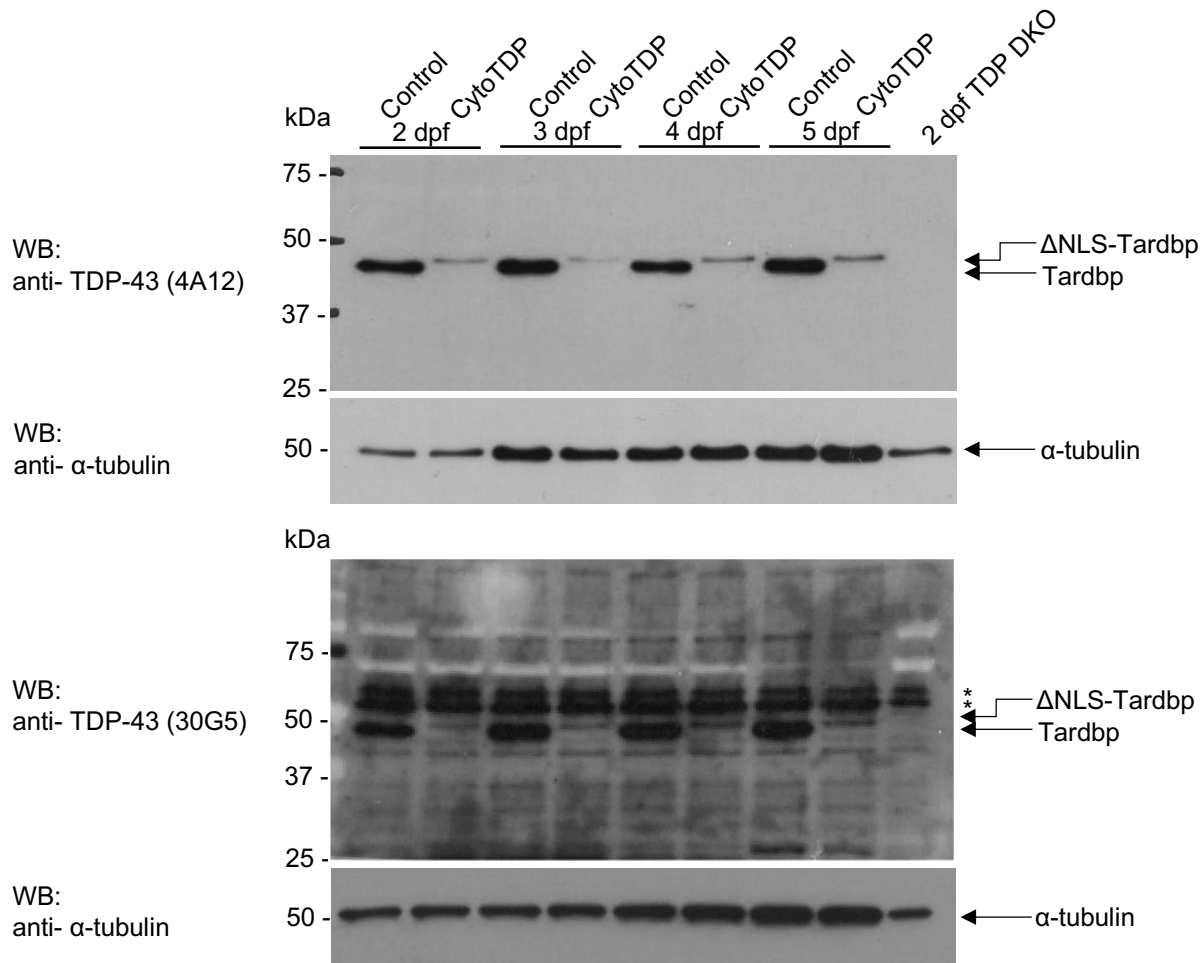

Supplement: Supplementary file 4 — Supplementary Material 4: SFigure 3. ΔNLS-Tardbp can be detected by 2 independent antibodies. (A) Schematic drawing shows different binding sites for the 4A12 and 30G5 Tardbp antibody. (B) Western blot analysis with Tardbp antibodies 4A12 and 30G5 reveal Tardbp levels in CytoTDP and Control embryos from 2 to 5 dpf. Asterics mark unspecific bands. α-tubulin serves as a loading control. [file 13024_2024_735_MOESM4_ESM.pdf]

**A**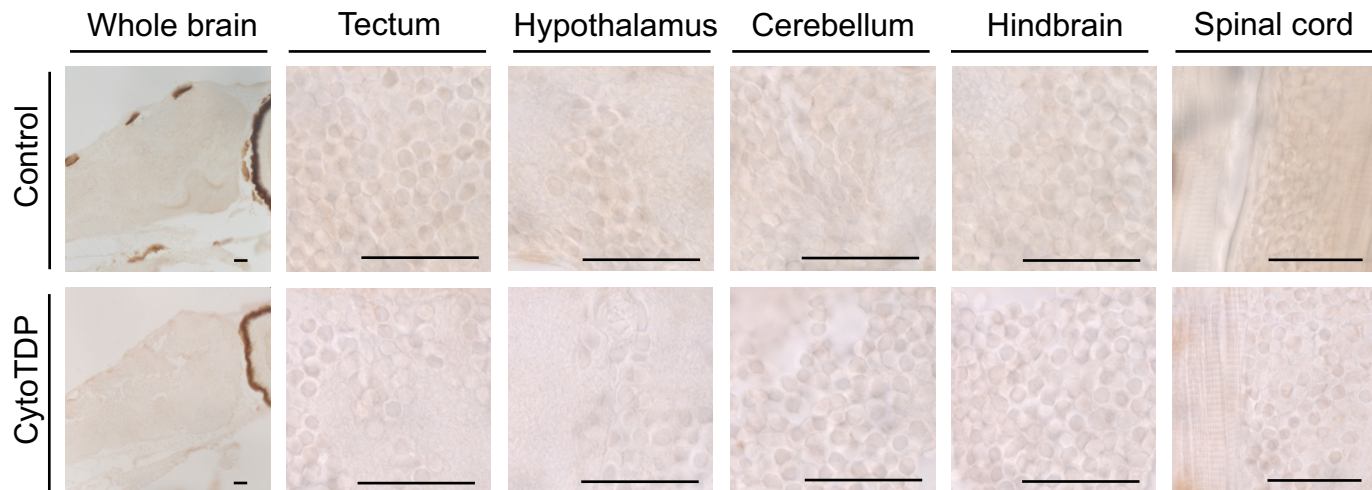**B**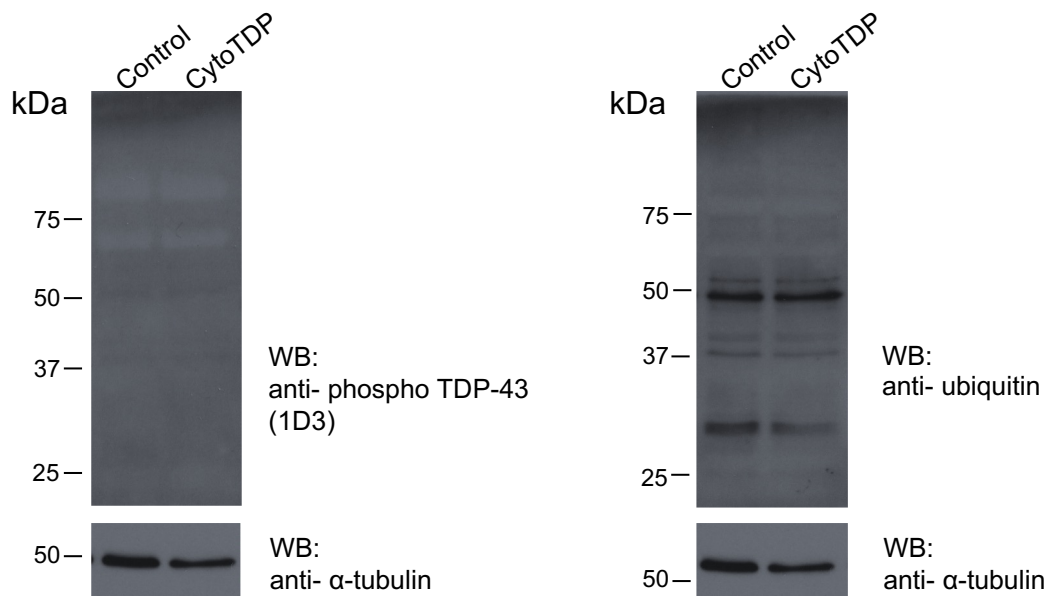

Supplement: Supplementary file 5 — Supplementary Material 5: SFigure 4. PhosphoTDP-43 staining, immunoblot and ubiquitin immunoblot show no obvious difference. (A) Immunohistochemical phosphoTDP-43 stainings of whole brain (including tectum, hypothalamus, cerebellum and hindbrain) and spinal cord paraffin sections of 5 dpf CytoTDP fish and Control fish show no differences between Control and CytoTDP fish. Scale bar = 100 μm. (B) Western blot analysis with a phospho TDP-43 antibody and an ubiquitin antibody for CytoTDP and Control larvae at 5 dpf. α -tubulin serves as a loading control. [file 13024_2024_735_MOESM5_ESM.pdf]

1.5 dpf

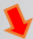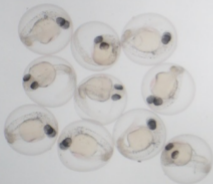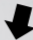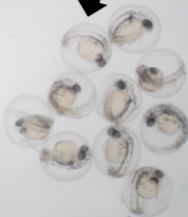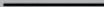

Supplement: Supplementary file 6 — Supplementary Material 6: SFigure 5. Decreased pigmentation phenotype in 1.5 dpf CytoTDP fish. Petri dish with sorted 1.5 dpf CytoTDP larvae according to their pigmentation phenotype (red arrow) and their siblings (black arrow). Scale bar = 2 mm. [file 13024_2024_735_MOESM6_ESM.pdf]

**A**

Control

CytoTDP

1.5 dpf

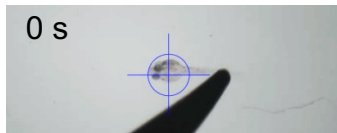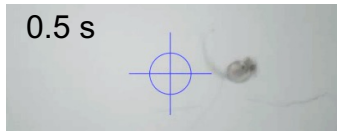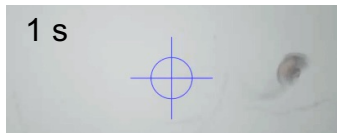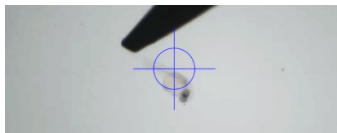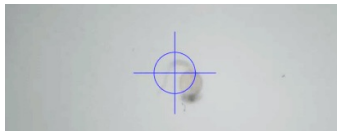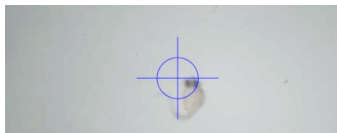**B**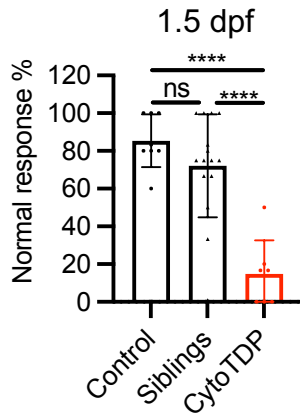

Supplement: Supplementary file 7 — Supplementary Material 7: SFigure 6. 1.5 dpf CytoTDP fish have reduced touch-evoked response. (A) Representative snapshot images of 1.5 dpf Control and zebrafish during touch-evoked response assay. (B) Quantification of normal response percentage for CytoTDP (red dots) and its siblings [Siblings (black arrow heads): tardbp ΔNLS/ + ; tardbpl -/-, Control (black dots): tardbp + / + ; tardbpl -/-] at 1.5 dpf. Kruskal–Wallis test was used for 3 group comparisons and Mann–Whitney test was used for 2 group comparisons. Error bars indicates ± interquartile range. Control n = 8, Siblings n = 15, CytoTDP n = 7. **** p < 0.0001, Control vs Siblings p = 0.2128, Control vs CytoTDP **** p < 0.0001, Siblings vs CytoTDP **** p < 0.0001. [file 13024_2024_735_MOESM7_ESM.pdf]

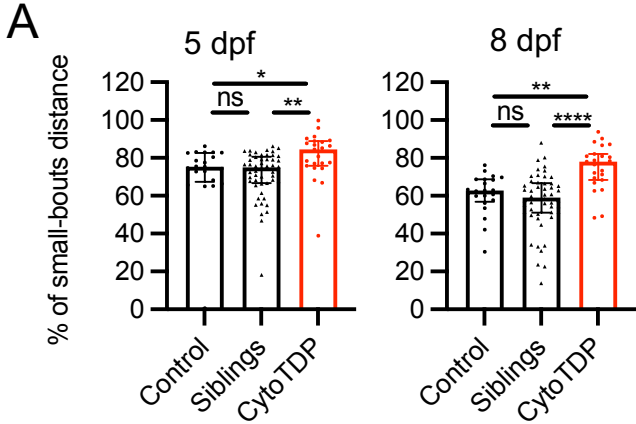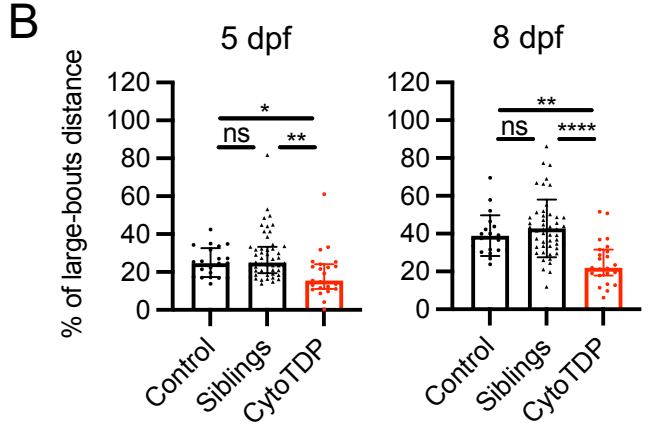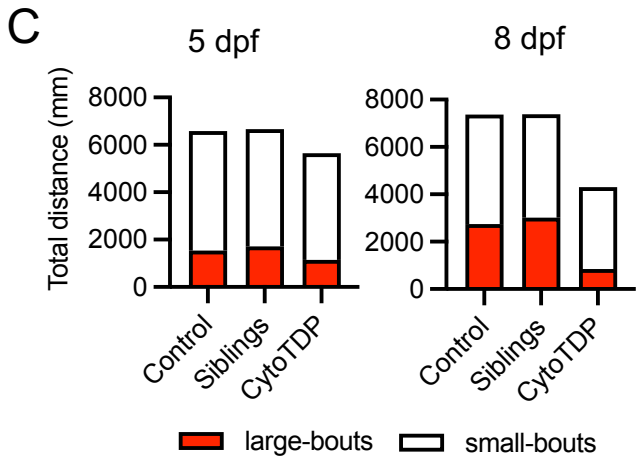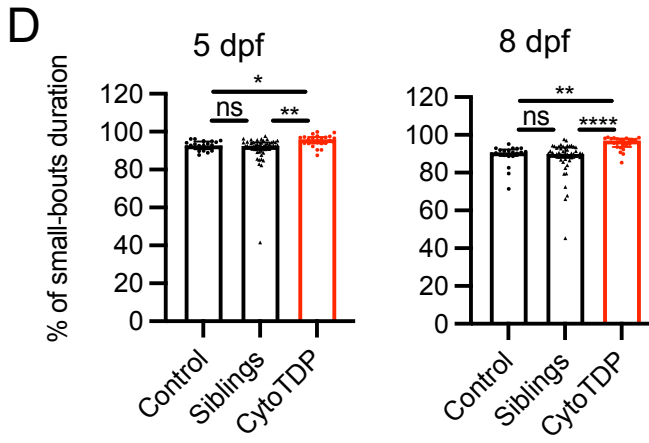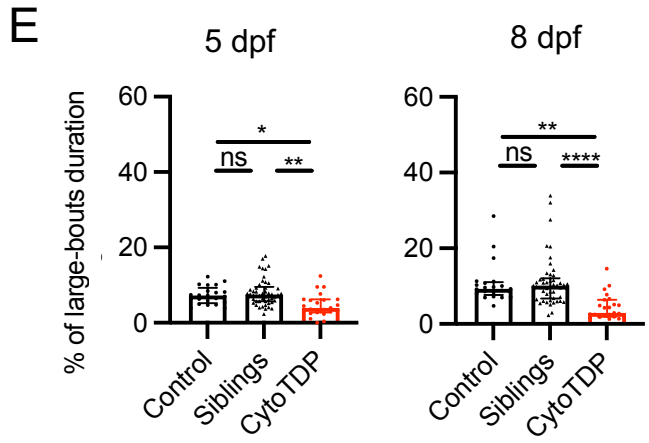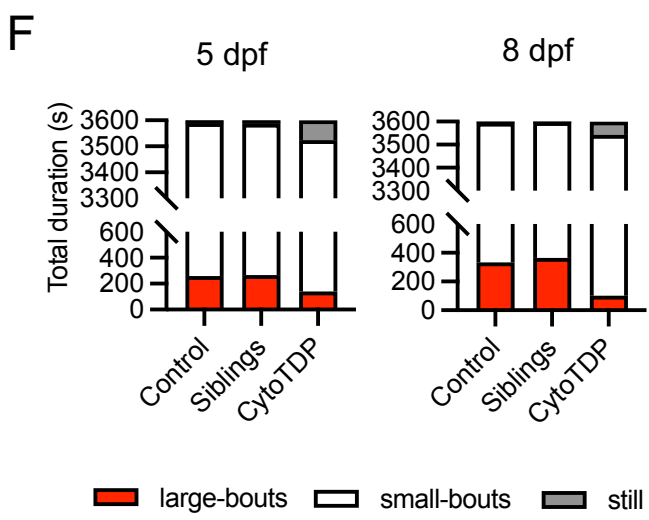

Supplement: Supplementary file 8 — Supplementary Material 8: SFigure 7. Detailed CytoTDP behaviour data. (A-B) Quantifications of small-bouts and large-bouts distance pencentage for CytoTDP (red dots) and its siblings [Siblings (black arrow heads): tardbp ΔNLS/ + ; tardbpl -/-, Control (black dots): tardbp + / + ; tardbpl -/-] at 5 dpf and 8 dpf. Kruskal–Wallis test was used for 3 group comparisons and Mann–Whitney test was used for 2 group comparisons. Error bars indicates ± interquartile range. Control n = 21, Siblings n = 50, CytoTDP n = 23. (A) Small-bouts distance pencentage [5 dpf (**p = 0.0015, Control vs Siblings p > 0.9999, Control vs CytoTDP * p = 0.0324, Siblings vs CytoTDP ** p = 0.0012); 8 dpf (****p < 0.0001, Control vs Siblings p > 0.9999, Control vs CytoTDP ** p = 0.0023, Siblings vs CytoTDP **** p < 0.0001)]; (B) Large-bouts distance percentage for CytoTDP and its siblings at 5 dpf and 8 dpf. [5 dpf (**p = 0.0015, Control vs Siblings p > 0.99, Control vs CytoTDP * p = 0.0375, Siblings vs CytoTDP ** p = 0.0011); 8 dpf (****p < 0.0001, Control vs Siblings p > 0.9999, Control vs CytoTDP ** p = 0.0023, Siblings vs CytoTDP **** p < 0.0001)]; (C) Stacked bar graph showing mean distribution of small-bouts distance (white bar) and large-bouts distance (red bar) for CytoTDP and its siblings at 5 dpf and 8 dpf. (D-E) Quantifications of small-bouts and large-bouts duration percentage for CytoTDP and its siblings at 5 dpf and 8 dpf. Kruskal–Wallis test was used for 3 group comparisons and Mann–Whitney test was used for 2 group comparisons. Error bars indicates ± interquartile range. Control n = 21, Siblings n = 50, CytoTDP n = 23. (D) Small-bouts duration pencentage [5 dpf (**p = 0.0015, Control vs Siblings p > 0.99, Control vs CytoTDP * p = 0.0324, Siblings vs CytoTDP ** p = 0.0012); 8 dpf (****p < 0.0001, Control vs Siblings p > 0.9999, Control vs CytoTDP ** p = 0.0023, Siblings vs CytoTDP **** p < 0.0001)] (E) Large-bouts duration percentage for CytoTDP and its siblings at 5 dpf and 8 dpf. [5 [file 13024_2024_735_MOESM8_ESM.pdf]
